# Biological Evaluation of Avocado Residues as a Potential Source of Bioactive Compounds

**DOI:** 10.3390/antiox11061049

**Published:** 2022-05-25

**Authors:** Alejandro Rojas-García, Eduardo Fuentes, María de la Luz Cádiz-Gurrea, Lyanne Rodriguez, María del Carmen Villegas-Aguilar, Iván Palomo, David Arráez-Román, Antonio Segura-Carretero

**Affiliations:** 1 Department of Analytical Chemistry, University of Granada, 18071 Granada, Spain; alejorogar@ugr.es (A.R.-G.); marivillegas@ugr.es (M.d.C.V.-A.); ansegura@ugr.es (A.S.-C.); 2 Thrombosis Research Center, Medical Technology School, Department of Clinical Biochemistry and Immunohematology, Faculty of Health Sciences, Universidad de Talca, Talca 3460000, Chile; edfuentes@utalca.cl (E.F.); lyrodriguez@utalca.cl (L.R.); ipalomo@utalca.cl (I.P.)

**Keywords:** avocado by-products, phenolic compounds, HPLC-MS, reactive oxygen species, enzyme inhibition, platelet aggregation

## Abstract

Avocado seed and peel are the main by-products from avocado industrialisation, and account for nearly 30% of fruit weight. Although they are usually discarded, their high phenolic content has been deeply associated with several nutritional and functional benefits. Thus, for a comprehensive analytical evaluation of both semi-industrial extracts, various steps have been developed: tentative characterisation and quantification of the phenolic composition using HPLC-ESI-qTOF-MS, determination of TPC and antioxidant activity by Folin–Ciocalteu, FRAP, TEAC and ORAC methods, evaluation of scavenging capacity against different ROS and measurement of the enzymatic inhibitory potential against potentially harmful enzymes. Finally, their bioactive potential was tested in a human platelet model where antiaggregatory activity was measured. Hence, 48 different compounds were identified, where flavonoids and procyanidins were the most representative groups. The higher TPC was found in avocado peel extract (190 ± 3 mg/g), which showed more antioxidant power and more capacity to decrease ROS generation than seed extract (60 ± 2 mg/g). In addition, both extracts showed enzymatic inhibition, especially against hyaluronidase, xanthine oxidase and acetylcholinesterase. Lastly, avocado peel was proven to inhibit platelet aggregation with significant results at 1, 0.75 and 0.5 mg/mL, where the extract showed reducing effects on agonists’ expression such as p-selectin or GPIIb/IIIa complex. These results demonstrate that both semi-industrial extracts—above all, avocado peel—have an interesting potential to be exploited as a natural by-product with antioxidant properties with multiple applications for the prevention of different pathologies.

## 1. Introduction

The avocado (*Persea americana* Mill.) is an important Central American fruit belonging to the *Lauraceae* family, produced mainly in tropical and subtropical areas, although it is currently cultivated throughout the world. Approximately, about six million tons of avocado are thought to be produced annually around the world [1], being the tropical fruit with the greatest production growth in recent years.

An average avocado fruit is composed of pulp (among 65–73%), peel (among 11–15%) and seed (among 16–20%) [2]. Recent studies have demonstrated that avocado fruit possesses high nutritional quality. The pulp is recognised for its high levels of vitamins, minerals, proteins and fibers, as well as high concentrations of unsaturated fatty acids and bioactive compounds such as carotenoids, hydroxybenzoic and hydroxycinnamic acids, procyanidins, condensed tannins and flavonoids, especially flavonols [3]. Nevertheless, peels and seeds have been also identified as interesting matrices due to their high content of bioactive compounds such as carotenoids, tocopherols and especially phenolic compounds [3]. Their main phenolics ever identified are derivatives of chlorogenic acid (caffeoylquinic and coumaroylquinic acids) and flavonoids (catechins, quercetin glycosides and procyanidins) [4]. Former studies have also demonstrated that avocado seed and peel show even higher amounts of phenolics than the pulp [5].

Because of this, avocado by-product phenolic compounds have already been associated with a host of health-related benefits: antioxidant, anti-inflammatory, anticarcinogenic, antiaging, antiaggregatory, antibacterial and antifungal activity, among others [6]. Due to antioxidant and anti-inflammatory activity, related to their ability to eliminate free radicals [7], these by-products are known to promote therapeutic effects against some human degenerative diseases associated with the presence of reactive oxygen species (ROS) and oxidative stress, including prevention and protection against neurodegeneration, cardiovascular and gastrointestinal failures and cancer development [1,8,9], or even against skin-aging-related issues, encouraging photoprotection from harmful UV sun rays, increasing of the wound-healing process and mitigation of skin hyperpigmentation [10,11,12].

Despite their proven bioactivity, these nonedible parts are commonly discarded. Annually, at least 1.6 million tons of avocado seeds and peels are estimated to be thrown away globally [1], turning them into a remarkable source of environmental contamination and provoking a huge wastage of nutritional value. Instead, these residues could be a low-cost onset to obtain a wide variety of phenolic acids and flavonoids with a high functional potential for the formulation of foods, nutraceuticals or cosmetic products [13].

In this context, and being aware of high economic value of avocado production, a comprehensive in vitro evaluation of the semi-industrial extract of *P. americana* Mill. by-products was carried out with the aim of highlighting the potential of seeds and peels as phytochemical sources, and their possible revalorisation from a preindustrial-scaling point of view. For this purpose, a tentative analytical characterisation/quantification of the phenolic composition was carried out, followed by an in-depth in vitro study evaluating parameters such as total phenolic content, antioxidant potential and free-radical scavenging activity, as well as the affinity of its phenolics with several enzymes involved in physiological phenomena by determining their inhibitory concentration (IC_50_). Finally, an evaluation of platelet antiaggregatory activity was performed to determine the anticoagulation capacity of avocado seed and peel, and thereby prove their cardiovascular benefits. In the present work, for the first time, a comprehensive enzymatic study was carried out on the main avocado by-products on a preindustrial scale, as well as their possible therapeutic applications (platelet aggregation), thus addressing their potential for the development of future nutraceuticals.

## 2. Materials and Methods

### 2.1. Chemical Reagents

For extractions and solutions, ultrapure water was obtained with a Milli-Q system Millipore (Bedford, MA, USA) and absolute ethanol was purchased from VWR chemicals (Radnor, PA, USA).

The following reagents were provided from the indicated suppliers: Sodium carbonate, acetic acid, TPTZ (2,4,6-tris(2-pyridyl)-s-triazine), sodium hydroxide and hydrochloridic acid were purchased from Fluka (Honeywell, NC, USA). Absolute ethanol and sulfuric acid were purchased from Riedel-de-Haën (Honeywell, NC, USA). Sodium hypochlorite solution EMPLURA was purchased from Merck (Darmstadt, Germany). NOC-5 was purchased from Chemcruz (Santa Cruz Biotech., Dallas, TX, USA). Gallic acid, Folin reagent, ABTS (2,2′-azinobis (3-ethylbenzothiazoline-6-sulphonate)), potassium persulfate, Trolox (6-hydroxy-2,5,7,8-tetramethylchroman-2-carboxylic acid), sodium acetate, ferric chloride, heptahydrate ferrous sulphate, fluorescein, AAPH (2,2’-azobis(2-amidinopropane) dihydrochloride), sodium phosphate monobasic and dibasic, DHR (dihydrorhodamine), DMF (dimethylformamide), potassium dihydrogen phosphate anhydrous, NADH (β-nicotinamide adenine dinucleotide), NBT (nitrotetrazolium blue chloride), PMS (phenazine methosulfate), DAF-2 (diaminofluorescein diacetate) tyrosinase inhibitor screening kit (colorimetric), Tris (tri(hydroxymethyl)aminomethane), acetylthiocholine iodide, 5.5-dithiobis-(2-nitrobenzoic acid), acetylcholinesterase from Electrophorus, Cayman’s xanthine oxidase fluorometric assay kit, neutrophil elastase colorimetric drug discovery kit, sodium chloride, hyaluronic acid, hyaluronidase from sheep testes, tricine, 1-10 phenantroline, collagenase from Clostridium histolyticum, FALGPA (*N*-[3-(2-furyl)acryloyl]-L-leucyl-glycyl-L-prolyl-L-alanine), adenosine diphosphate (ADP), thrombin receptor activating peptide 6 (TRAP-6), sodium citrate 3.2% and phosphate buffer solution (PBS) were purchased from Sigma-Aldrich (St. Louis, MO, USA). Collagen was purchased from Havertown, PA, USA.

### 2.2. Extraction of Avocado Agroindustrial By-Products

Fresh dark avocado fruits of the variety ‘Hass’ were donated by the commercial group La Caña, Miguel García Sánchez e Hijos, S.A. (Motril, Spain) to NATAC Biotech. S.L. (Cáceres, Spain) in order to obtain a preindustrial extract from avocado by-products. Complete avocado seeds and peels were manually separated and cleaned under continuous flow of tap water. Then, a 3-cycle solid–liquid extraction (maceration) at 50–70 °C (seeds and peels, respectively) using a hydroalcoholic mixture (EtOH 60%-peels and 70%-seeds) was carried out. Ethanol/water is considered as a favourable solvent in the extraction of polar substances such as phenolic compounds, which does not have toxic effects on humans and is environmentally friendly (GRAS solvent) [7]. Each cycle was performed on 20 kg of seeds or peels mixed with 200 L of extractant over 2 h. Subsequently to decantation, microfiltration and liquid-extract concentration, a biconical rotary vacuum dryer was used to obtain the final dry extracts at 50–60 °C (seeds and peels, respectively), with a certain amount of silicon dioxide to promote peels (4%) and seeds (10%) drying. Once well-dried, both extracts were ground and sieved, turning them into samples of an average size of 2 mm. The extraction efficiency from extracts obtained were 15.7 ± 0.9 g dry extract per 100 g of raw material for peel, and 14.6 ± 1.2 g dry extract per 100 g of raw material. The material was stored at room temperature and protected from light until their analysis.

### 2.3. HPLC-ESI-TOF-MS Analysis

Avocado seed and peel extracts at 5000 mg/L were analysed using high-performance liquid chromatography (HPLC), specifically an ACQUITY UPLC H-Class System (Waters, Milford, MA, USA) coupled to an electrospray-quadrupole time-of-flight mass spectrometer (ESI-qTOF-MS, Synapt G2, Waters Corp., Milford, MA, USA) working in negative-ion mode over a range from 50 to 1200 *m/z*. The separation was performed in a ACQUITY UPLC BEH Shield RP18 Column, 130 Å, 1.7 µm, 2.1 mm × 150 mm at a flow rate of 0.7 mL/min using volume injection of 10 μL.

The mobile phases were (A) acidified water with 1% of acetic acid (*v/v*), and (B) acetonitrile. The following multistep linear gradient was used in order to achieve efficient separation: 0.0 min [A:B 99/1], 2.33 min [A:B 99/1], 4.37 min [A:B 93/7], 8.11 min [A:B 86/14], 12.19 min [A:B 76/24], 15.99 min [A:B 60/40], 18.31 min [A:B 2/98], 21.03 min [A:B 2/98], 22.39 min [A:B 99/1] and 25.0 [A:B 99/1]. To acquire mass spectrum, two parallel scan functions were performed, switching among them rapidly. Of both scans, one was operated at low collision energy in the gas cell (4 eV) and the other at an elevated collision energy (MS^E^ energy linear ramp: from 20 to 60 eV). Leu-enkephalin was injected for mass calibration continuously. Other parameters were as follows: source temperature 100 °C; scan duration 0.1 s; resolution 20,000 FWHM; desolvation temperature 500 °C; desolvation gas flow 700 L/h; capillary voltage 2.2 kV; cone voltage 30 V; cone gas flow 50 L/h.

Finally, data obtained were processed and visualised using MZmine 2.53 open-source software and Sirius 4.4.29. In addition, by contrasting information provided by the software with the literature available for both avocado and other species belonging to Lauraceae family, the compounds’ characterisation was achieved. Literature search for published spectral information was carried out by using SciFinder^®^.

### 2.4. Quantification of Individual Phenolic Compounds by HPLC-ESI-qTOF-MS

The phenolic compounds identified in the extracts were tentatively quantified using calibration curves of the respective reference compounds, all of them obtained with a good linearity (R^2^ > 0.99) by plotting the standard concentration as a function of the peak area obtained from HPLC-ESI-qTOF-MS analyses [14]. For this purpose, a pattern mix was prepared as from stock solutions (500 mg/L) diluted to concentrations of 0.488–31.25 mg/L (catechin, procyanidin B, verbascoside, myricetin-3-glucoside, quercetin, quercetin glucoside and quinic acid). In the case that reference compounds were not relatable enough, the quantification of some compounds was performed using structurally related substances, provided that the phenolic compound standard had an aglycon moiety similar to those present in the test sample. Table 1 summarises the analytical parameters for the different phenolic compounds present in avocado extracts.

### 2.5. In Vitro Assays for Bioactive Determination of Phenolic Compounds in Avocado By-Products

All undermentioned assays performed were adapted to a 96-well polystyrene microplate, and the absorbance measurement was carried out on a Synergy H1 Monochromator-Based Multi-Mode Micro plate reader (Bio-Tek Instruments Inc., Winooski, VT, USA).

#### 2.5.1. Phytochemical Analysis and Evaluation of In Vitro Antioxidant Potential

(a) Total Phenolic Compound Content Assessment by Folin–Ciocalteu Method

Total phenolic content was determined following Folin–Ciocalteu (F-C) method, reported by Gurrea-Cadiz et al., with some modifications, and using different proportions of by-product extract (100, 200 and 500 mg/L) [15]. Total phenol content was expressed as microgram of gallic acid equivalent (GAE) per milligram of dry extract (DE) (μg GAE/mg DE). Measurements were made in triplicate.

(b) Ferric Reducing Antioxidant Power (FRAP)

This method, carried out by Al-Duais et al., with some modifications, is used to measure the reducing antioxidant power of the different proportions of avocado wastes extracts (100, 200 and 500 mg/L) in comparison with a calibration curve constructed with ferrous sulfate (FeSO4·7H2O). Results were expressed as μmol of iron equivalent per DE (μM Fe (II)/g). Measurements were made in triplicate.

(c) Trolox Equivalent Antioxidant Capacity (TEAC)

TEAC was in vitro measured as the reducing activity of extracts at different concentrations (100, 200 and 500 mg/L) against ABTS * +, a way of calculating antioxidant capacity based on ability to scavenge that radical. As Re et al. reported, Trolox was used as the standard at concentrations from 0.5 to 15 μM [16]. The results were expressed as mmol Trolox equivalent per grams of DE. Measurements were made in triplicate.

(d) Oxygen Radical Absorbance Capacity (ORAC)

To assay the capacity of the extracts to scavenge peroxyl radicals, a validated ORAC method by Huang et al., was carried out [17]. ORAC values were calculated using a regression equation between the Trolox concentration and the area under the fluorescence decay curve. The results are expressed as μmol Trolox equivalents per grams of DE. Measurements were made in triplicate.

#### 2.5.2. Evaluation of Free Radical and ROS Scavenging Potential

All free-radical scavenging assays were performed and adapted according to Gomes et al. and Pinto et al. [18,19].

(a) Scavenging Ability of Superoxide Anion Radical (O_2_ *)

Superoxide anions were generated by a nonenzymatic PMS-NADH system, and the scavenging activity was evaluated using a colorimetric methodology in the microplate reader based on the reduction of NBT into a purple-coloured diformazan as result of the reaction with superoxide anions at 560 nm. The sample concentration providing 50% inhibition (IC_50_) was achieved by interpolating this inhibition percentage against extract concentrations.

(b) Scavenging Ability of Nitric-Oxide Radical (NO *)

Nitric-oxide anions were generated by the presence of NOC-5, and 4,5-diaminofluorescein (DAF-2) was used as the fluorescent probe applied [18,19].

An incubation at 37 °C was needed, and a fluorescence measure at 485–528 nm for excitation emission was performed. Results are expressed as IC_50_ values obtained as aforementioned.

(c) Scavenging Ability of Hypochlorous Acid (HOCl)

The method was based on the fluorescent HOCl-induced oxidation of DHR to rhodamine [18,19]. Results are expressed as the inhibition, in IC_50_, of this oxidation of DHR inducted by HOCl.

#### 2.5.3. Evaluation of Enzymatic Inhibition Potential

(a) Inhibition of Acetylcholinesterase (AChE)

AChE inhibitory activity was measured by using a photometric colour-based assay described by Ellman et al., with certain modifications [20]. The reaction starts with acetylthiocholine (ATCI) acting as the substrate and being cleaved by AChE to form thiocholine, which in turn reacts with DTNB to give the yellow 5-thio-2-nitrobenzoate anion. The enzyme activity was measured by following the rate of production of thiocholine, thus the increase in yellow colour produced.

The ratio of colour production was measured every minute at 405 nm. Tests were carried out in triplicate, and the IC_50_ was calculated using different avocado-extract concentrations.

(b) Inhibition of Tyrosinase

The test was carried out utilizing the “Tyrosinase Inhibitor Screening Kit (Colorimetric)” (Sigma-Aldrich, USA). Tyrosinase catalyses the oxidation of tyrosine, producing a chromophore that can be detected at 510 nm. Thus, stablishing an inhibition control using kojic acid and an enzyme control using only tyrosinase, every avocado sample tyrosinase inhibition activity could be measured by calculating chromophores production.

Briefly, solvents were added to the microplate according to its role (sample, inhibitor or enzyme control). Later, enzyme and substrate solution were added into each well and the measurement took place at 510 nm. Tests were performed in triplicate, and the IC_50_ was calculated using different avocado-extract concentrations.

(c) Inhibition of Xanthine Oxidase (XO)

Avocado by-products’ XO inhibitory activity was measured using the kit “Cayman’s Xanthine Oxidase Fluorometric Assay Kit” (Cayman Chem. Ann Arbor, MI, USA). The method is based on the production of a highly fluorescent compound named resorufin as of oxidation of hypoxanthine by XO releasing H_2_O_2_. As the reaction takes place, resorufin fluorescence can be easily analysed with an Ex/Em wavelength of 535/587 nm.

Two different buffers were used for this methodology: assay buffer, needed to prepare the assay cocktail; and sample buffer, used for sample and enzyme dilutions. Once the wells were loaded with enzyme/sample, the microplate was incubated for 10 min at 37 °C and then the cocktail was added. Finally, the fluorescence measurement was performed, taking data every 2 min over 20 min. All measurements were made in triplicate, and results are expressed as IC_50_.

(d) Inhibition of Elastase

The elastase inhibition assay was performed according to the previously reported method from Pinto et al., but considering several modifications [21]. This assay relies on hydrolysis of substrate MeOSuc-Ala-Ala-Pro-Val-pNa by elastase in order to release a certain amount of p-nitroaniline, which is determined with a maximum absorbance at 405 nm. In brief, the substrate, inhibitor (elastatinal) and enzyme were prepared in buffer (pH 7.25). Subsequently, each well was refilled with the prepared reactives according to whether it was a sample or not. After 30 min of incubation at 37 °C, the absorbance of solutions was measured at 405 nm. All measurements were made in triplicate, and results are expressed as IC_50_.

(e) Inhibition of Hyaluronidase (HYALase)

The HYALase inhibitory activity measurement was performed following—with some adjustments—the method described by Nema et al. [22,23]. The procedure is based on evaluation of intensity loss from transmitted light due to particles suspended in it to obtain HYALase activity. These particles are derived from the enzymatic reaction of hyaluronic acid (HYAL), which leads to di and monosaccharides and small HYAL fragments.

Consequently, absorbance was measured at 600 nm. Tests were carried out in triplicate, and the IC_50_ was calculated using different avocado-extract concentrations.

(f) Inhibition of Collagenase

Finally, inhibitory effect against collagenase was measured following the methodology conducted by Kumar et al., but also modifying certain parameters [22]. The assay requires Tricine buffer (pH 7.5), substrate FALGPA, and collagenase from *Clostridium histolyticum*. This colorimetric method is based on the measurement of the degradation of FALGPA after incubation with the enzyme. Post-incubation, the absorbance was measured at 335 nm. Tests were carried out in triplicate, and the IC_50_ was calculated using different avocado-extract concentrations.

### 2.6. Evaluation of Platelet Antiaggregatory Potential

Extracts were lyophilised and dissolved in phosphate-buffered saline (PBS) for the antiaggregatory studies as a pretreatment.

#### 2.6.1. Obtaining Human Platelets

The blood was obtained from healthy volunteers, free of nonsteroidal anti-inflammatory drugs (NSAIDs), who previously signed informed consent according to the protocol approved by the Scientific Ethics Committee of the University of Talca in accordance with the Declaration of Helsinki [24]. Blood was collected by venepuncture of the forearm with citrate tubes. The citrate tubes were centrifuged at room temperature for 10 min (240 g) to obtain platelet-rich plasma (PRP). Subsequently, the blood was centrifuged again for 10 min at 800 g to obtain platelet-poor plasma (PPP), which is used to adjust the platelet concentration of PRP (200 × 109 platelets/L) [25]. Platelet counts were performed on a Haematology Counter (Mindray BC-3000 Plus Haematology Counter, Kobe, Osaka, Japan).

#### 2.6.2. Studies on Platelet Aggregation

The antiplatelet activity of the avocado extracts was evaluated by turbidimetry, using a lumi-aggregometer (Chrono-Log, Haverton, PA, USA). The lyophilised extracts of avocado peel and pit were diluted in phosphate-buffered saline and were incubated for 6 min at room temperature with PRP (200 × 109 platelets/L) at a concentration of 1 mg/mL first. The negative control, 100% platelet aggregation, was obtained by incubating the PRP with PBS. Platelet aggregation was stimulated by adding ADP (4 µM), TRAP-6 (10 µM) or collagen (1 µg/mL), for 6 min at 37 °C. Results were obtained as mean ± SEM of 6 volunteers provided by with AGGRO/LINK software (Chrono-Log, Havertown, PA, USA). Platelet aggregation (PA) inhibition was calculated as [26]:

$$\mathrm{PA}\;\mathrm{Inh}.\;(\%)=100\;-\;((\frac{\mathrm{PA}\;\mathrm{of}\;\mathrm{avocado}\;\mathrm{extracts}}{\mathrm{PA}\;\mathrm{of}\;\mathrm{the}\;\mathrm{negative}\;\mathrm{control}})\;\times \;100)$$

Avocado seed and peel extracts were evaluated at different concentrations (1 mg/mL, 0.75, 0.5, 0.25 mg/mL and 0.1 mg/mL) on platelet aggregation induced by ADP, TRAP-6 and collagen.

#### 2.6.3. Activation Markers and Platelet Secretion

Platelet purity was determined by flow cytometry. PRP was added to an Eppendorf and anti-CD61-FITC was added. The expression of P-selectin and activation of GPIIb/IIIa was assessed by flow cytometry (BD FACSLyric) as previously described with some modifications [27]. The PRP was incubated with avocado extracts (peel or seed) or control (vehicle) for 10 min at 37 °C. Platelet aggregation was stimulated with ADP (4 µM), TRAP-6 (10 µM) and collagen (1 µg/mL) for 6 min at 37 °C. The samples were incubated with CD62-PE (P-selectin) or PAC-1-FITC (GPIIb/IIIa) for 30 min at room temperature in the dark. Platelet populations were selected according to cell size using scatter (FSC) versus side scatter (SSC) and CD61 positivity to distinguish it from electronic noise, as described by other authors [28]. Measurements were made on platelets from six healthy volunteers.

#### 2.6.4. Statistical Analysis

The data obtained are presented as the mean ± standard error of the mean (SEM) of the individual experiments and analysed using Prism 6.0 software (GraphPad Inc., San Diego CA, USA). Platelet-inhibition results were analysed by ANOVA and Tukey’s post hoc test to determine significant differences between samples [29].

## 3. Results and Discussion

### 3.1. Characterisation of Avocado Seed and Peel Extracts by HPLC-ESI-qTOF-MS

In order to interpretate and identify to some extent the diversity of available bioactive phenolic compounds from avocado seed and peel extracts, a preliminary analytical characterisation by using HPLC-ESI-qTOF-MS was performed. Thus, the representative base peak chromatograms (BPCs) from both extracts are shown in Figure S1. The compounds’ identification comprised interpretation of the accurate mass spectra provided by qTOF-MS and the confirmation by using the information previously reported in literature [5,30,31,32,33,34]. All proposed phytochemical compounds were numbered according to their elution order and gathered in Table 2, where different mass spectral data are recollected, such as their retention time, *m/z*, molecular formula, compound name and quantification values expressed as mean ± standard deviation in mg of analyte per gram of dry extract (DE).

The analysis allowed the tentative identification of a total of 49 different compounds, among which 42 compounds were exclusively found in avocado peel. Organic and phenolic acids, flavonoids, catechins, procyanidins, phenylpropanoids, lignans, sugars, fatty acids and other polar compounds were identified during the characterisation.

In the present study, a total of 18 compounds were identified and characterised in avocado seed extract. According to the phenolic structure, the classification consists of organic acids, such as quinic and citric acids; phenolic acids derived from hydroxycinnamic acids, such as chlorogenoquinone isomers or galloylquinic acid; proanthocyanins, specifically procyanidin A trimer isomers, rare alcoholic sugars such as perseitol and penstemide, and even an important phytohormone such as abscisic acid derivative, involved in regulation of seed development [30,34]. The total phenolic amount calculated was 14 ± 1 mg per g of DE for seed extract, taking into account that several phenolic compounds were impossible to be quantified since calibration ranges excluded their results.

Avocado peel extract was composed mainly of monomeric, glycosylated and condensed flavonoids. This group is regarded as the major peel phenolic group (20 compounds), mostly formed by glycosylated flavonol derivatives. Catechin/epicatechin were also identified, but it was impossible to be distinguished. Some other flavonoid subtypes were found: flavanols such as isorhamnetin and kaempferol; flavones such as luteolin; and flavonolignans such as isolariceresinol [32]. Almost every identified flavonoid was part of a heteroside, constituting the nonglycosylated fragment conjugated to distinct glycosylated moieties. Quercetin showed more glycosylated derivatives than any other, with 11 compounds, 17 taking into account isomers. [35].

On the other hand, several A- and B-type procyanidins, whose chemical structure is based on the presence of (epi)catechin units linked by single bonds, were found. Thus, peaks 10, 11 and 12 corresponded to B dimer, trimer and tetramer at *m/z* = 577.4579, *m/z* = 865.1994 and *m/z* = 1153.2576, respectively, and peaks 15 and 25 corresponded to A trimer and dimer at *m/z* = 863.1796 and *m/z* = 575.1190, respectively.

Organic- and phenolic-acid groups are comprised of almost the same as avocado seed: quinic and citric acid, and chlorogenoquinone isomers. A phenylpropanoid was found for the first time in this characterisation: lariciresinol feruloyl glucopyranoside. Finally, some alcoholic sugars and glucosylated-acid derivatives were also found: perseitol, penstemide and hydroxyabscisic acid glucoside. The total phenolic amount calculated was 66 ± 2 mg per gramme of DE, almost 5 times more than the seed content.

Several compounds have been tentatively identified for the first time in avocado seed and peel matrixes, such as peaks 5, 6 and 8 from seed extract, and peaks 6–8, 32, 44 and 46 from peel extract. The majority of these compounds have been identified before in other vegetal matrixes. In addition, it was checked that molecular formula and *m/z* matched [36,37,38]. Quinones are structures known for being responsible for the brown colour after an enzymatic oxidation of the fruit, so it could be thought that the avocado fruit was starting to suffer these reactions from the presence of chlorogenoquinones [39].

Regarding the avocado colour, it has always been related to pigment concentration and the ripening stage. While the green colour of non-Hass avocados are due to the high presence of chlorophyll in the peel, the characteristic dark colour of Hass avocados are highly caused by anthocyanins [40]. In turn, ripeness regulates phytochemical composition of the fruit and its by-products, along with conditions of growth and variety of avocado [41]. Therefore, as the ripeness increases, phenolic content and antioxidant capacity in seed seems to also increase, thus being also related with the fruit colour [42]. Moreover, the colour and texture of avocado peel changes in types and amounts of phenolics, e.g., all structures formed by (epi)catechin units decrease their levels at early maturation [43].

### 3.2. Evaluation of Total Phenolic Content

As a previous step to measure the antioxidant capacity of avocado by-products extracts, the total phenolic content (TPC) was assessed. Methods used to its measurement are hard to be compared due to the fact that all of them are commonly extremely dependent on the reaction conditions and the substrates or products, so not all methods yield the same values for activity [44]. In such circumstances, Folin–Ciocalteau was chosen as the method to be performed. The procedure’s main disadvantage is a weak accuracy, since is based on a quite generic reduction reaction, allowing a lot of molecules to interfere in the assay [15]. Nevertheless, is widely spread as an approximate assay for semiquantitative phenolic compounds from plant extracts due to its simpleness, reproducibility and robustness [45]. Furthermore, it was performed using a 96-well microplate spectrophotometry methodology, originally devised for food samples.

The obtained values for each extract are shown in Table 3. Avocado peel and seed extracts were redissolved in an 80/20 (*v/v*) solution of ethanol/water. On the basis of the DE, the total phenolic content in avocado-seed extract was 60 ± 2 mg GAE per g, while avocado-peel extract was 190 ± 3 mg GAE per g. According to previous reports, avocado seed and peel showed higher phenolic content than average pulp [46,47]. Likewise, seed extract showed lower phenolic content than that observed in avocado-peel extract. Their comparison has been scarcely addressed; a significantly higher content of bioactive compounds in avocado peel extracts has been reported compared to that from its seed [4,7]. This difference in phenolic content could be attributed to distinct exposures to the environmental stress factors [48]. As Oboh et al. reported, stress factors provoke intense synthesis of phenolic compounds to prevent oxidative damage of plant cellular structures. The seed, which is protected by the edible portion of the fruit, is less exposed to such stress factors as, for instance, ultraviolet rays from sunlight, so the phenolic synthesis is lower.

In this work, TPC values for avocado extracts were compared to other studies previously carried out: the peel extract exerted higher TPC than reported by Trujillo-Mayol et al. [49] (92.5 mg GAE/g DE) and Rodríguez-Carpena et al. [50] (from 32.93 to 89.97 mg GAE/g DE)—both also ‘Hass’ variety. This last study also reported TPC values for the seed, from 16.99 to 60.82 mg GAE/g DE, close to resulting values from this assay. On the other hand, higher TPC from ‘Hass’ hydroethanolic extract was reported by Dibacto et al. (813 ± 12 mg GAE/g DM) [51]. Differences among values are due to many factors, such as type of extractant, extraction method, fruit size, ripening stage, etc. In comparison to other tropical fruit by-products, results are uneven: Alañón et al., studied mango kernel seed and reported TPC for different varieties from 78 to 80.5 mg GAE/g DM [52]. Lopes dos Santos et al., reported for guava fruit TPCs from 0.81 to 34.14 mg GAE/g DM [53]. Cádiz-Gurrea et al., reported 964.05 ± 82.29 mg GAE/g DM for grape seed though their extracts were commercial and 95% enriched [14].

### 3.3. Evaluation of Antioxidant Capacity Using TEAC, FRAP and ORAC

Antioxidant potential is involved with the ability to protect a biological system against the harmful effect of oxidative processes. These antioxidants are fundamental for the preservation of the biological system from reactive species [54]. The intricacy in the reactions that antioxidants exert to perform their activity hinders the determination of its biological power in a sample. Moreover, since antioxidant capacity measured by a specific assay only reflects the conditions applied in that assay, to use one type of reaction would be inefficient at predicting every oxidative detail of a system [55]. Therefore, it is usual to perform various methodologies based on different mechanisms through phenolic compounds exerting their antioxidant power depending on their structure [44]. HAT reactions are based on the transfer of a hydrogen atom, while the SET mechanism is based on the transfer of a single electron. Regarding some of the most used methodologies, ORAC tests the capacity of extracts to quench radicals through a HAT mechanism, and TEAC and FRAP evaluate the ability of neutralise through SET reactions. Their combination could offer more approachable results and a precise representation of the global antioxidant capacity of avocado samples.

In agreement with a higher phenolic content, avocado peel also reported higher FRAP, TEAC and ORAC values than avocado seed (Table 3). Phenolic compounds have been reported as responsible for the antioxidant activity of herbal extracts [54], which could be demonstrated by the existing correlation among TPC and antioxidant values in by-products extracts: avocado peel stands out as a better antioxidant by electron-transfer-based and hydrogen atom-transfer-based mechanisms. To understand this remarked antioxidant activity, characterisation results from avocado peel were revised, showing major diversity of phenolic molecules of high molecular weight and higher polymerisation than those found in seed, such as procyanidins dimers, trimers and tetramers. These features have been formerly related to significant antioxidant power [5,14,56].

In addition, antioxidant activity strongly depends on chemical structure: phenolic category, arrangement of hydroxyl groups and other functional groups bound to aromatic rings, as well as the conformation of the ring itself, affect their power [57,58]. Based on this, and supported by TEAC results, Rice-Evans et al. stated that the most impressive bioactive and antioxidant profile is provided by quercetin and its structural features [59,60]. Knowing that this compound was only identified in avocado peel in large quantities forming several types of glycosidic bounds, in addition to the presence of some phenolic acids with ortho-diphenol skeleton and several highly polymerised procyanidins, could confirm the quenching ability and reducing power of avocado peel.

The FRAP, TEAC and ORAC values in peel and seed of ‘Hass’ *P. americana* variety have been extensively reported in the literature. Tremocoldi et al. reported FRAP and TEAC values for Hass peel and seed (1175.1 and 656.9 µmol Fe/g DE; and 791.5 and 645.8 µmol TE/g DE, respectively), which compared with ours showed a downward trend, especially the peel sample [7]. The same occurred with Vieira Amado et al., with TEAC results of 313.46 and 17.28 µmol TE/g DE for Hass peel and seed, respectively; and Kosinska et al., with TEAC results of 161 and 94 µmol TE/g DE for Hass peel and seed, respectively, versus 1510 and 496 µmol TE/g DE reported in this study [46,61]. Even ORAC values for peel and seed from Kosinska et al. were lower than results presented here (0.47 and 0.21 mmol/g DE versus 1.78 and 0.57 mmol/g DE, respectively). Nevertheless, other studies reported greater results, such as Segovia et al., who, using the same extraction conditions, noticed TEAC and ORAC values of 645.8 µmol TE/g DE and 616.48 mmol/g DE for Hass seed, successively [62]. These differences among antioxidant values are associated to a host of factors that affect the recovering of bioactive compounds, most notably (I) the use of different extraction techniques, one of the most important issues [30], (II) the use of solvents with distinct natures and proportions, and (III) the differences in avocado by-products extracts’ origins and supply sources.

Castañeda-Valbuena et al. reported TEAC values for mango seed and peel that ranged from 1 to 5 mmol TE/g DE, and from 1 to 4.4 mmol TE/g DE, respectively [63]. Cádiz-Gurrea et al. reported FRAP values of 6.47 ± 0.47 and 3.95 ± 0.21 mmol Fe^2+^/g DE, TEAC values of 6.1 ± 0.8 and 4.19 ± 0.14 mmol TE/g DE, and ORAC values of 8.62 ± 0.73 and 6.99 ± 0.5 mmol TE/g DE for grape seed and *Theobroma cacao*, respectively [14]. Morais et al., also performed FRAP on tropical samples, obtaining 0.0105 and 0.0895 mmol Fe^2+^/g DE for papaya seed and peel, respectively, and 0.1193 and 0.0603 mmol Fe^2+^/g DE for passion fruit seed and peel, respectively [64]. From revising literature, no extract—except for those enriched—showed higher values than those exerted by avocado extracts.

In the present study, the avocado by-products’ extracts were provided from a semi-industrial source, and all the results reviewed from literature were prepared on a laboratory scale. Pilot plant processes are less efficient than laboratory processes, as they maintain significant pureness and stability parameters. Therefore, in comparison, it would be comprehensible that our avocado peel and seed extracts would offer lower values than the laboratory-prepared samples [65].

### 3.4. Evaluation of Free Radical and ROS Scavenging Potential

In average corporal biochemical processes, the endogenous generation of free-radical species is quite common, e.g., the superoxide radical is constantly produced by mitochondrial and microsomal electron-transport chains or by reductions or certain enzymes such as xanthine oxidase [7]. Therefore, radicals’ concentration in the organism should be considerably—despite its toxicity—harmful to several biological compounds. An excessive generation of these species, or a large exposure to exogenous oxidizing chemical agents promotes oxidative stress, which has been closely related to various disease conditions such as cancer, asthma, diabetes or cardiovascular pathologies, among others [66,67]. Free radicals are highly reactive species capable of damaging even DNA, proteins and lipids in the cells.

The superoxide radical anion (O_2_^•−^) is an extremely reactive compound triggered by reduction of oxygen by a single electron produced during certain catalytic enzymatic roles. Its scavenging is particularly important, due to the fact that it is ubiquitous in aerobic cells, and despite its mild reactivity is a potential precursor of aggressive hydroxyl radical (HO^•^) [7,68]. The nitric-oxide radical (NO^•^) is widely created by living organisms: endothelial cells, macrophages, neurons, etc. [69]. It is involved in modulation and regulation of certain physiological processes, reacting with singlet oxygen to produce, through intermediates, stable products of nitrate and nitrite related to blood and ‘haemo’ phenomena [70]. However, nitric oxide is also regarded as an important mediator of acute and chronic inflammation, easily reacting with superoxide anion to form potent oxidizing molecules that provoke cellular damage. Thus, an excess concentration is linked with corporal detriment, displaying multiple cytotoxic effects that would trigger a host of different diseases such as inflammation, cancer or atherosclerosis, among others [67,71]. Hypochlorous acid (HOCl) is generated in neutrophils by reactions of chlorides with hydrogen peroxide. Its endogenous production constitutes an important defence mechanism against microorganisms [7]; nevertheless, it also promotes haemolysis on erythrocytes, being associated with several pathological processes such as atherosclerosis [58,72].

Table 3 shows the results of avocado by-product extracts obtained after evaluation of the radical-scavenging capacity of three average endogenous reactive species whose corporal excessive concentration develops serious physiological damage. Generally, avocado peel resulted in the best ROS scavenger extract, with almost the lowest IC_50_ values, although avocado seed exhibited more hypochlorous-acid-scavenging capacity than peel (2.3 mg/L vs 7.1 mg/L, respectively). Table 4 also shows positive controls tested to compare to our results: for radical neutralisation, gallic acid (GA) and epicatechin (EPI) were chosen as positive scavengers. Avocado-peel extract showed worse results for O_2_^•−^ scavenging (380 ± 69 mg/L vs. 50 ± 3 mg/L, GA, and 70 ± 5 mg/L, EPI) and almost the same results for NO^•^ scavenging (1.90 ± 0.09 mg/L vs. 1.4 ± 0.3 mg/L, GA, and 0.87 ± 0.02 mg/L, EPI). Avocado seed showed better results than GA for HOCl scavenging (2.3 ± 0.1 mg/L vs. 3.8 ± 0.3 mg/L, respectively), which brings interest to the extract. Except for O_2_^•−^, avocado peel exerted a significantly high antiradical activity, with similar magnitude to those showed by GA and EPI.

Plant phenolics can act as antioxidant agents by different mechanisms. The first one, radical scavenging, was formerly mentioned and evaluated, highlighting avocado peel’s ability to neutralise free-radical species. Another antioxidative mechanism, metal chelation, is also closely related to this matter [73]. Catechol-moiety flavonoids and phenolic acids are also efficient at chelation of transition metals, which have the role of natural enhancers of ROS formation in living organisms [68]. Andjelkovic et al. observed that hydroxycinnamic acids (chlorogenic and caffeic acids) performed the best complex formation thanks to their ortho-diphenol chelating domain [73].

Taking together metal chelation activity and the formerly mentioned radical scavenging ability, the antioxidant activity of avocado peel and seed is totally proven. The peel is pointed out as a significant better source of antioxidant substances than avocado seed, based on reported values and a wide-ranging diversity of phenolic compounds qualitatively determined by HPLC-ESI-qTOF-MS. However, both are practical and feasible options in food, pharmacological and cosmetic industries [1].

After reviewing reported literature, almost nothing was found about testing the radical-scavenging ability of ‘Hass’ avocado by-products’ extracts, specially about hypochlorous acid. Tremocoldi et al. addressed this issue and obtained, from Hass seed to peel, 52 and 70 mg/L for superoxide radical, and 5.2 and 6.7 mg/L for hypochlorous acid. In comparison, although our avocado extracts displayed somewhat less superoxide radical-scavenging capacity than reported, our peel extract exhibited nearly half the seed value. On the other hand, both hypochlorous-acid-scavenging studies bore some similarity [7]. Other studies did exhibit lower results than ours, such as Alagbaoso et al., with antioxidant activity against superoxide anion values between 1500 and 3400 mg/L for avocado seed, or Kamaraj et al., which reported nitric-scavenging activity of 79.05 mg/L and superoxide-scavenging activity of 103.05 mg/L for avocado peel [66,69]. However, the variety of fruit was not specified in any of the latter two studies, and since no direct comparison could be made among the different species, it is difficult to assess with the results reported. Concerning the differences among experiments, it could be thought that the composition of by-products is a determining factor in the total scavenging ability of the extract. Therefore, observing the identification of phenolic compounds, quercetin is highlighted for being located only in avocado peel, and for a remarkable bioactivity against species such as O_2_^•−^ and NO^•^ [74,75]. The higher presence of quercetin in avocado peel probably promotes its ability for blocking radical species, making its IC_50_ lower than the seed one.

Regarding other tropical species, *Limonia acidissima* L. showed a range from 60 to 125 mg/L as IC_50_ of nitric-oxide-scavenging radical activity [76]. The stem bark of mango showed an IC_50_ scavenging activity against HOCl of 400 mg/L [77]. In nontropical fruit by-products, shells from *Castanea sativa* were tested for O_2_^•−^ and HOCl scavenging capacity, showing 49.42 ± 0.41% at 500 mg/L, and 50% at 1.57 ± 0.10 mg/L, respectively [19]. In conclusion, avocado extracts (especially peel) show excellent properties at scavenging radical species in comparison to other fruit species.

### 3.5. Evaluation of Enzymatic Inhibition Capacity

ROS can be originated by intrinsic or extrinsic factors, with first referring to oxidative/nitrosative stress and altered metabolism, and the second referring to long exposures to exogenous harmful agents, e.g., UV radiation. In this sense, the excess of reactive species leads to many detrimental conditions for human body, in which the activation of different enzymes, such as acetylcholinesterase, tyrosinase, xanthine oxidase, elastase, hyaluronidase and collagenase is closely linked [21].

Acetylcholinesterase (AChE) enzyme is responsible for acetylcholine (ACh) regulation, a significantly important compound involved in nerve-impulse transmission between cells (cholinergic synapses). In its presence, AChE rapidly breaks down ACh into choline and acetate, thus promoting neurological disorders related to cholinergic transmission deficit: Alzheimer’s disease, senile dementia, ataxia and myasthenia gravis. Tyrosinase enzyme is responsible for the physiological synthesis of melanin, the production of which in human skin is known as a primordial defence mechanism against UV radiation. However, overproduction and unrestricted accumulation could lead to the formation of epidermal pigmentation, considered the first sign to skin aging and some deleterious disorders related: melasma, age spots, flecks, ephelides and sites of actinic damage. Xanthine oxidase (XO) enzyme is a dehydrogenase responsible for catalysing hypoxanthine to xanthine, and subsequently to uric-acid oxidation. However, under oxidative-stress conditions, XO is transformed in an oxidase, responsible for dangerous superoxide-radical production and causing many pathological diseases, such as gout, hyperuricemia, hepatitis, carcinogenesis and aging. Elastase, hyaluronidase (HYALase) and collagenase enzymes are responsible for, respectively, elastin, hyaluronic acid (HYAL) and collagen regulation, the main substances in the extracellular matrix (ECM) and closely related in order to maintain its structural organisation, structure integrity and elasticity. These enzymes, under ROS overproduction, promote skin-aging phenomena through fibre-network depletion, leading to the loss of skin elasticity, decrease in holding-water capacity and consequent skin disorders such as skin sagging, wrinkle formation and eczemas, among others [78,79,80,81,82].

Therefore, the coherent blocking of these key enzymes would improve their beneficial state on human health. This effect can be induced by plant phenolics with strong antioxidant power. Table 3 shows results obtained from the enzyme-inhibition assessment.

According to their IC_50_ or IC_30_ values, the peel exhibited a higher degree of inhibition of XO and elastase, while the seed showed it for AChE and HYALase, in spite of the fact that it showed the lowest degree of all inhibition values for elastase, and no inhibitory effect at all on tyrosinase. Several assays previously carried out stated that hydroxyl groups from flavonoids and phenolic compounds could interact with the backbone or with some functional group of enzymes; or that interactions between the enzyme and the antioxidant result in conformational changes that finally lead to unfunctional enzymes. Enzyme inhibitory capacity is directly related to the existence of certain phenols and flavonoids such as epicatechin, catechin or quercetin [82,83,84]. Hence, the inhibitory activity of avocado by-products extracts could be explained again by their phenolic high concentrations. For example, quercetin activity against collagenase has been reported before, and being aware that only avocado peel harbours quercetin and derivatives, it could explain its lower IC_50_ [85]. The power this flavonol exerts against tyrosinase and elastase activity has also been noted [86]. Nevertheless, the slight anti-elastase potential from both extracts may be due to the attachment between main flavonoids with sugars to positions 3 or 7, which seems to downregulate the anti-elastase activity of the aglycone [87]. In the case of anti-acetylcholinesterase activity, previous reports highlight avocado leaf extract as interesting neuroprotector solutions; a quantification was performed and the levels of chlorogenic acid stood out, which may be the reason why both our extracts showed cholinergic potential [88].

Comparing our results with controls from Table 4, some interpretations can be made: positive controls from tyrosinase, elastase and AChE exerted higher inhibitory activity than both avocado extracts. Drugs as positive controls, such as physostigmine, offer extremely specific activity against certain targets, so the comparison among it and extracts could be confusing. Nevertheless, the remaining enzymes were more inhibited by avocado seed and/or peel than the controls: in the case of collagenase, knowing that 4500 ppm of phenanthroline were needed to inhibit 83 ± 2%, avocado seed and peel needed 104 and 81 ppm, respectively, to inhibit a 50%. It could be thought that avocado extracts exert higher inhibitory activity than the control. In the case of HYAL, once the IC_50_ value was known, the final order was GA = EPI < AP < AS; both extracts highlighted as better HYALase inhibitors than positive controls. Similarly, in the case of XOD, the order was EPI < AS < AP, also offering higher activity than the chosen control. These studies confirm the possibilities that both extracts offer depending on the target.

Concerning the industrial potential of these extracts to regulate enzyme production, the quite limited number of studies published in literature is noteworthy. In the case of AChE, Oboh et al. only reported avocado (unknown variety) leaf and seed extract values, 33.72 and 27.93 mg/mL, respectively, against our seed value of 0.0583 mg/mL (IC_50_) [48]. Tyrosinase was also found in literature only performed for avocado seed (unknown variety), offering an IC_50_ of 93.02 mg/L, oppositely to our nonexistent result [12]; other inhibition values were also reported by Fawole et al., from seven cultivars of pomegranate peel, in ranges from 3.66 ± 0.11 to 98.66 ± 0.12 mg/L (IC_50_) [89]. XOD inhibition assay was only found on Persea leaves, with a resulting IC_50_ of 63.39 mg/L [90]. In the case of elastase and HYALase, no literature was found concerning avocado or its by-products. Samejima et al. performed the anti-elastase and anti-HYALase assay on guava leaf extract, another tropical fruit, with IC_50_ results between 17.7 and 42.5 mg/L for elastase; and between 377 and 875.4 mg/L for hyaluronidase [91]. Finally, regarding anticollagenase activity, Figueroa et al. reported an inhibition of 43.7% at 150 mg/L of avocado ‘Hass’ peel, less active in comparison to our result (50% at 81 mg/L) [35]. Mangiferin obtained from *Mangifera indica* leaves, peels and barks reported inhibition results against elastase (IC_50_ 58.9 ± 3.9 mg/L) and collagenase (IC_50_ 107.09 ± 3.19 mg/L) [92]. Comparisons highlight avocado peel for its use inhibiting AChE, collagenase, HYALase and XO.

### 3.6. Evaluation of Platelet Antiaggregatory Activity

Cardiovascular diseases (CVDs) are considered as the leading cause of death worldwide, with ischemic heart disease and stroke being the two main causes of death, as pointed out by the World Health Organization [93]. In addition, these pathologies have been deeply associated with an increase in platelet function. Platelets are small anucleated blood cells responsible for maintaining a balance between activatory and inhibitory signalling pathways for haemostasis and thrombosis phenomena. Nevertheless, excessive platelet activation is a decisive factor enhancer of CVDs disorders such as hypertension, diabetes and atherosclerosis, among others [94].

The platelet activation process, composed of different cellular signalling pathways, allows the involvement of a host of different substances and species, such as platelet agonists or ROS. They act as second messengers by stimulating the arachidonic-acid metabolism and phospholipase C pathway. Excessive production enhances oxidative stress, which would regulate several components of thrombosis, including platelet activation. All this cellular stress displays a critical role in CVDs [95]. Agonists such as collagen, von Willerbrand factor (VWF), adenosine diphosphate (ADP), thrombin or thromboxane 2 (TXA2), are platelet-activation stimulators. They are responsible for inducing signalling cascades that result in conformation changes in αIIb3 integrin, creating an activated complex with improved affinity to fibrinogen and enhanced adhesive properties [96].

In this sense, it is necessary to search for new strategies to modulate platelet activity. The consumption of fruits and vegetables with high phenolic-compound content has formerly been profoundly reported because of their important role exerting platelet antiaggregatory activity [27,97]. This effect on platelet function seems to be possible at different levels: due to structure-dependent interferences (number of hydroxyl groups, C4 carbonyl substituted, C3 hydroxylated and a B ring with catechol moiety); inhibition of ROS production, reducing oxidative burst, modulating certain pathways or blocking agonistic substances [94]. Accordingly, platelet antiaggregatory assessments could be performed through several mechanisms; in this study, the measure was carried out, in the first place, using the inhibition of ADP, collagen and TRAP-6 technique in order to choose the most significant powerful extract capable of inhibiting the action of these different thrombus agonists. Subsequently, the inhibition of ADP, collagen or TRAP-6-stimulated P-selectin secretion and GP IIb/IIIa activation were also evaluated, and both events related to platelet activation and adhesion: the first one is a cohesive molecule released during the platelet activation and promoted the thrombus formation, and the latter is an integrin complex whose main function is the reception of different agonists that regulate the activation of platelets [98,99].

In the first place, the antiplatelet activity of avocado seed and peel extracts was evaluated by turbidimetry at 1 mg/mL. Platelet aggregation was stimulated with ADP (4 µM), TRAP-6 (10 µM) and collagen (1 µg/mL), as it is shown in Table 5. It was observed that avocado peel and seed extracts inhibited agonist-stimulated platelet aggregation in study.

Avocado-peel extract showed greater antiplatelet potential than avocado-seed extract against the evaluated agonists, TRAP-6, ADP and collagen. The antiplatelet activity of avocado-peel extract was higher when platelet aggregation was stimulated with ADP and collagen: 78 ± 2% and 55 ± 2%, respectively. This effect was less marked compared to TRAP-6, 42 ± 1. Meanwhile, the avocado-seed extract only showed significant inhibition of platelet aggregation against collagen, 45 ± 2%. Considering the positive control adenosine (10µM), avocado peel stands out as a great antiaggregatory extract, especially when promoted by ADP and collagen.

The extracts that showed higher antiplatelet potential were selected, with a percentage of platelet inhibition above 50%. The concentration-dependent antiplatelet activity of avocado-peel extract induced by ADP and collagen was also studied.

**Figure 1:**
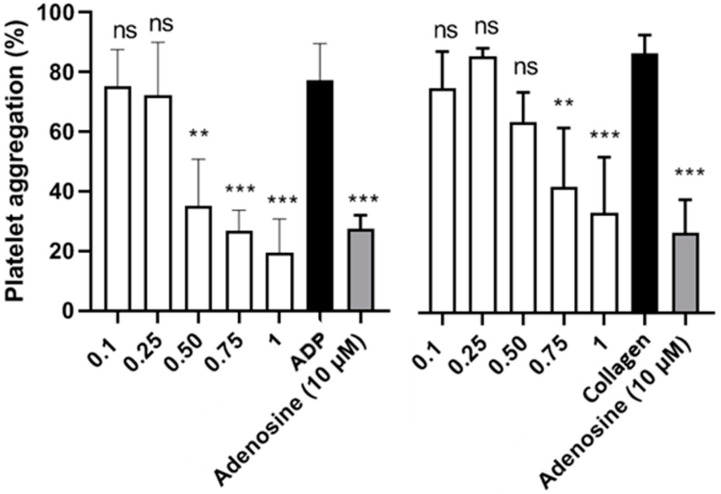
Study of platelet aggregation of avocado peel and seed extract induced by collagen and ADP. The PRP was previously incubated with vehicle or avocado extract (0.1, 0.25, 0.50, 0.75 and 1 mg/mL). After 3 minutes of incubation at 37 °C, it was stimulated with the agonist to initiate platelet aggregation for 6 minutes. The negative control is in the absence of the extracts. Bar graph indicates maximum aggregation expressed as a percentage (mean ± SEM; *n* = 6). Differences between groups were analysed by ANOVA using Dunnet’s post hoc test. *** *p* < 0.001 and ** *p* < 0.01, denote statistically significant differences compared to the vehicle; ns: nonstatistical difference with respect to the vehicle (PBS).

In this sense, the obtained results (Figure 1) show that the antiplatelet activity of avocado-peel extract is concentration-dependent, being able to inhibit collagen-stimulated platelet aggregation up to 0.5 mg/mL compared to the control. Nevertheless, when platelet aggregation was induced with ADP, the antiplatelet potential remained significant at higher concentrations, 1 mg/mL and 0.75 mg/mL, while at the lowest concentration the activity decreased markedly. Actually, avocado peel showed higher anticoagulant activity than adenosine, even at 0.75 mg/mL.

**Figure 2:**
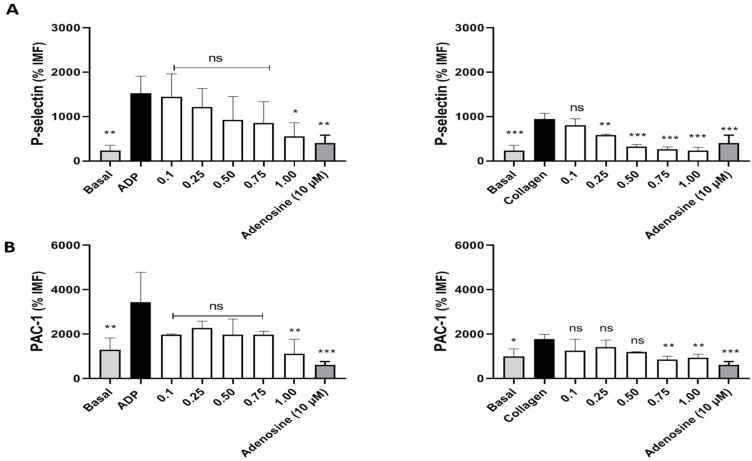
Effect of avocado-peel extract on the expression of platelet-activation markers. (**A**) Effect on P-selectin expression; (**B**) Effect on PAC-1 expression. Platelets were stimulated with ADP or Collagen. Platelets were identified as a CD61 + population. Statistical analysis was performed by ANOVA (Dunnet’s test). * *p* < 0.05, ** *p* < 0.01 and *** *p* < 0.001 vs. Vehicle (PBS) vs. activated control (agonist) (*n* = 5).

On the other hand, other studies were conducted to determine the effect of avocado-peel extract on platelet activation, p-selectin expression and activation of GP IIb/IIIa (Figure 2). Platelet activation was stimulated with the agonists ADP and collagen, since in these conditions relevant antiplatelet effects of avocado peel extract were observed. When platelets were stimulated with ADP, only at 1 mg/mL p-selectin expression was inhibited. However, when platelet activation was stimulated with collagen, the expression of p-selectin was significantly reduced to a concentration of 0.25 mg/mL with respect to the activated condition (maximum expression of p-selectin). Comparing to positive control, the extract works better in a collagen-agonist model at concentrations from 0.5 to 1 mg/mL.

Finally, the evaluation of the activation of GP IIb/IIIa showed that the avocado-peel extract reduced the platelet activation induced by ADP and collagen (activated state), with this effect being more powerful when activated with collagen. Activation of GP IIb/IIIa decreased at higher concentrations of avocado extract, 0.75 and 1 mg/mL. These results suggest that avocado-peel extract inhibits platelet activation by reducing P-selectin secretion and GP IIb/IIIa activation.

## 4. Conclusions

The use of HPLC-ESI-qTOF-MS enabled the identification of 48 different compounds in avocado seed and peel semi-industrial extracts—some of them never identified before in the avocado by-products matrix—and the tentative quantification of 38 phenolic compounds from different families. Glycosylated flavonols, hydroxycinnamic acids and procyanidins are the most representative groups in both samples, mostly in avocado peel.

Avocado-peel extract showed quite greater antioxidant effects than avocado seed, in terms of TPC, capacity to donate electrons (FRAP and TEAC) and transfer hydrogen atoms (ORAC), and ability to scavenge reactive species (·O_2_ and NO). In addition, results of enzyme assay revealed that compounds from avocado peel were effective inhibitors for every enzyme tested, especially for hyaluronidase, xanthine oxidase and acetylcholinesterase. Regarding avocado-seed extract, it also shown significant phenolic content, antioxidant capacity and enzyme inhibition (HOCl, hyaluronidase, xanthine oxidase).

Moreover, platelet antiaggregatory effect has been proven to be performed by avocado-peel extracts, which means a reduction in thrombus formation and other cardiovascular benefits. The mechanism through this effect is exerted must be studied more profoundly, but it seems that phenolic compounds and other bioactives from avocado peel are able to reduce the effect of agonists, inhibit adhesive molecules and inactivate protein complexes closely involved in platelet aggregation. Therefore, due to differences among phytochemical compositions and the biological potential, avocado by-products can be considered as an excellent source of antioxidants that could be employed for therapeutical needs.

Regarding the extraction procedure, although emergent extraction techniques such as ultrasound or microwave-assisted extractions are very popular for the obtainment of phytochemicals through environmentally friendly procedures, these methods have been used almost exclusively on a laboratory scale. This study aims to provide new information about how avocado wastes could be treated from a preindustrial scale-up point of view for those industries that generate many tons of waste per year and want to invest on revalorisation. The main disadvantage of emergent techniques for industry is that they are very expensive and require rather high temperatures (> 130 °C) for their application, which are major limitations for industrial equipment and make these methodologies not very feasible to an industrial scale-up.

Consequently, in order to ease industrial application, SLE was chosen as the extraction method. As a future perspective, knowing the high number of greener extraction techniques than SLE that currently exist, research and studies must focus on applying them in a practical way for industrial scale-up, thus reducing operation time and pollutant emission without a disproportionate rise in costs.

## Figures and Tables

**Table 1:** Quantification data of identified phenolic compounds from avocado seed and peel.

Standard	LOD (µg/mL)	LOQ (µg/mL)	Calibration Range (mg/L)	Calibration Equations	R^2^
Quinic acid (1)	0.04	0.14	(0.977–7.813)	y = 1099.56 x − 21.48	0.999
Quinic acid (2)	0.04	0.14	(3.906–31.25)	y = 2155.60 x − 5059.59	0.99
Procyanidin B1 (1)	0.37	0.95	(0.977–3.906)	y = 336.61 x − 39.08	0.999
Procyanidin B1 (2)	0.37	0.95	(3.906–15.625)	y = 857.10 x − 1913.35	0.998
Catechin	0.46	1.43	(1.953–31.25)	y = 857.50 x − 748.37	0.999
Quercetin	0.08	0.19	(0.488–31.25)	y = 3177.80 x − 2495.07	0.997
Quercetin glucoside	0.09	0.29	(0.488–31.25)	y = 2820.85 x + 688.34	0.993
Myrecetin-3-glucoside	0.18	0.48	(0.488–15.625)	y = 1289.08 + 737.99	0.995
Verbascoside	0.09	0.29	(0.488–15.625)	y = 2199.92 x − 213.06	0.998

Limit of detection (LOD) and quantification (LOQ), patterns used to quantify for each compound, linear equations and the coefficient of variation (R2).

**Table 2:** Identification and quantification of phytochemical compounds in avocado seed and peel extracts with ethanol/water by HPLC-ESI-qTOF-MS.

Peak	RT (min)	[M-H]^−^	Mol. Formula	Compound	Content (mg/g DE)
**Seed**					
1	0.46	343.0352	C_14_H_16_O_10_	Galloylquinic acid	4.0 ± 0.1
2	0.62	211.0805	C_7_H_16_O_7_	Perseitol	NQ
3	0.68	191.0546	C_7_H_12_O_6_	Quinic acid	3.3 ± 0.4
4	0.78	191.0539	C_6_H_8_O_7_	Citric acid	1.8 ± 0.3
5	4.28	597.2170	C_28_H_38_O_14_	Picraquassioside C	NQ
6	4.41	351.0695	C_16_H_16_O_9_	Chlorogenoquinone isomer 1	NQ
7	5.38	443.1907	C_21_H_32_O_10_	Penstemide	NQ
8	6.05	351.0705	C_16_H_16_O_9_	Chlorogenoquinone isomer 2	NQ
9	6.38	387.1643	-	Unknown	NQ
10	8.66	441.1741	C_21_H_30_O_10_	Hydroxyabscisic acid glucoside	NQ
11	8.81	863.1824	C_45_H_36_O_18_	Procyanidin A trimer isomer 1	NQ
12	9.87	863.1804	C_45_H_36_O_18_	Procyanidin A trimer isomer 2	2.5 ± 0.3
13	10.12	863.1821	C_45_H_36_O_18_	Procyanidin A trimer isomer 3	2.7 ± 0.5
14	11.29	472.1606	-	Unknown	NQ
15	12.11	461.2371	-	Unknown	NQ
16	15.07	329.2321	C_18_H_34_O_5_	Trihydroxyoctadecenoic acid	NQ
17	16.01	329.2330	C_18_H_34_O_5_	Trihydroxyoctadecenoic acid	NQ
18	17.68	315.2522	C_14_H_20_O_8_	Hydroxy salidroside	NQ
**Total phenolic amount**					**14 ± 1**
**Peel**					
1	0.46	343.0360	C_14_H_16_O_10_	Galloylquinic acid	3.1 ± 0.1
2	0.67	191.0544	C_7_H_12_O_6_	Quinic acid	5.7 ± 0.7
3	0.78	191.0542	C_6_H_8_O_7_	Citric acid	2.0 ± 0.3
4	0.83	545.0979	C_14_H_20_O_7_	Trigalacturonic acid	NQ
5	5.37	443.1907	C_21_H_32_O_10_	Penstemide	NQ
6	6.02	351.0711	C_16_H_16_O_9_	Chlorogenoquinone isomer 1	NQ
7	6.02	173.0445	C_21_H_32_O_10_	Shikimic acid	NQ
8	6.27	351.0717	C_16_H_16_O_9_	Chlorogenoquinone isomer 2	NQ
9	6.80	289.0704	C_15_H_14_O_6_	(Epi)catechin	7 ± 2
10	7.19	577.4579	C_30_H_26_O_12_	Procyanidin B dimer	NQ
11	7.89	865.1994	C_45_H_38_O_18_	Procyanidin B trimer isomer 1	2.1 ± 0.2
12	8.22	1153.2635	C_60_H_50_O_24_	Procyanidin B tetramer isomer 1	1.31 ± 0.10
13	8.55	865.1959	C_45_H_38_O_18_	Procyanidin B trimer isomer 2	NQ
14	8.64	441.1741	C_21_H_30_O_10_	Hydroxyabscisic acid glucoside	NQ
15	8.85	863,1796	C_45_H_36_O_13_	Procyanidin A trimer	2.8 ± 0.3
16	8.97	1153.2576	C_60_H_50_O_24_	Procyanidin B tetramer isomer 2	2.1 ± 0.2
17	9.11	521.2003	C_26_H_34_O_11_	Isolariciresinol glucid derivative	NQ
18	9.23	625.1390	C_27_H_30_O_17_	Quercetin diglucoside isomer 1	3.9 ± 0.3
19	9.33	625.1389	C_27_H_30_O_17_	Quercetin diglucoside isomer 2	0.7 ± 0.1
20	9.57	565.2265	C_28_H_38_O_12_	Quercetin derivative isomer 1	1.48 ± 0.09
21	9.81	595.1292	C_26_H_28_O_16_	Quercetin arabinosyl glucoside isomer 1	3.4 ± 0.3
22	9.90	595.1311	C_26_H_28_O_16_	Quercetin arabinosyl glucoside isomer 2	0.6 ± 0.2
23	10.00	609.1468	C_27_H_30_O_16_	Quercetin rutinoside isomer 1	1.79 ± 0.09
24	10.03	505.2083	C_23_H_22_O_13_	Quercetin acetylglucoside	1.10 ± 0.07
25	10.08	575.1190	C_30_H_24_O_12_	Procyanidin A dimer isomer 1	2.2 ± 0.2
26	10.33	575.1185	C_30_H_24_O_12_	Procyanidin A dimer isomer 2	2.0 ± 0.2
27	10.36	595.1302	C_26_H_28_O_16_	Quercetin arabinosyl glucoside isomer 3	0.53 ± 0.07
28	10.49	463.0859	C_21_H_20_O_12_	Quercetin glucoside isomer 1	1.5 ± 0.1
29	10.62	463.0849	C_21_H_20_O_12_	Quercetin glucoside isomer 2	0.55 ± 0.07
30	10.73	579.1335	C_26_H_28_O_16_	Luteolin pentosyl hexoside	2.1 ± 0.2
31	10.93	565.1187	C_28_H_38_O_12_	Quercetin derivative isomer 2	0.52 ± 0.02
32	11.00	299.0178	C_15_H_8_O_7_	Norwedelactone	NQ
33	11.08	609.1468	C_27_H_30_O_16_	Quercetin rutinoside isomer 2	9.6 ± 0.7
34	11.18	447.0891	C_21_H_20_O_11_	Quercetin rhamnoside isomer 1	0.52 ± 0.05
35	11.32	433.0750	C_20_H_18_O_11_	Quercetin arabinoside	<LOQ
36	11.48	447.0919	C_21_H_20_O_11_	Quercetin rhamnoside isomer 2	<LOQ
37	11.72	447.0905	C_21_H_20_O_11_	Quercetin rhamnoside isomer 3	0.79 ± 0.09
38	11.77	285.0393	C_15_H_10_O_6_	Luteolin	0.36 ± 0.02
39	11.89	579.1340	C_26_H_28_O_15_	Quercetin xylosyl rhamnoside	1.96 ± 0.05
40	11.98	593.1517	C_27_H_30_O_15_	Kaempferol glucosyl rhamnoside	1.89 ± 0.09
41	12.01	341.1376	C_20_H_22_O_5_	Obovatifol	NQ
42	12.06	315.0488	C_16_H_12_O_7_	Isorhamnetin	1.1 ± 0.1
43	12,53	575.1195	C_30_H_24_O_12_	Procyanidin A dimer isomer 3	<LOQ
44	12,81	563.1403	C_26_H_28_O_14_	Apigenin glucoside derivative	<LOQ
45	13.04	585.2366	C_24_H_42_O_16_	Glycosidic derivative	NQ
46	13.70	697.2519	C_36_H_42_O_14_	Lariciresinol feruloyl glucopyranoside	0.38 ± 0.02
47	14.37	327.2166	C_18_H_32_O_5_	Fatty acid	NQ
48	14.54	383.1488	-	Unknown	NQ
49	14.57	285.0391	C_15_H_10_O_6_	Kaempferol	0.38 ± 0.07
50	15.03	329.2313	C_18_H_34_O_5_	Trihydroxyoctadecenoic acid	NQ
51	15.97	329.2317	C_18_H_34_O_5_	Trihydroxyoctadecenoic acid	NQ
**Total phenolic amount**					**66 ± 2**

RT: retention time; DE: dry extract.

**Table 3:** Evaluation of total phenolic content, antioxidant capacity, radical scavenging and enzymatic inhibition of avocado by-product extracts.

Methodology	AS Extract	AP Extract
**TPC (mg GAE/g DE)**	60 ± 2	190 ± 3
**FRAP (mmol Fe^2+^/g DE)**	0.393 ± 0.005	1.427 ± 0.002
**TEAC (μ** **mol TE/g DE)**	496 ± 2	1510 ± 1
**ORAC (mmol TE/g DE)**	0.57 ± 0.02	1.78 ± 0.05
**HOCl (mg/L) ^1^**	2.3 ± 0.1	7.1 ± 0.2
**·O_2_ (mg/L) ^1^**	656 ± 4	380 ± 69
**·NO (mg/L) ^1^**	4.5 ± 0.6	1.90 ± 0.09
**AChE (mg/L) ^1^**	58.3 ± 0.8	67 ± 4
**Tyrosinase (mg/L) ^1^**	-	158 ± 7
**XO (mg/L) ^1^**	6.3 ± 0.7	4 ± 1
**Elastase (mg/L) ^2^**	790 ± 50	475 ± 2
**HYALase (mg/L) ^1^**	7 ± 1	8 ± 1
**Collagenase (mg/L) ^1^**	104 ± 8	81 ± 1

AS: avocado seed; AP: avocado peel; FRAP: ferric reducing antioxidant power assay; TEAC: Trolox equivalent antioxidant capacity; ORAC: oxygen radical absorbance capacity; GAE: gallic acid equivalent; DE: dry extract; TE: Trolox equivalent. Data are means ± standard deviation (*n* = 3). ^1^ Inhibitory concentration at 50%. ^2^ Inhibitory concentration at 30%.

**Table 4:** Positive controls from radical scavenging and enzymatic inhibitions.

Methodology	GA	EPI	PHY	PHE	ELA	KA
**HOCl (** **mg/L) ^1^**	3.8 ± 0.3	0.18 ± 0.01	X	X	X	X
**·O_2_ (mg/L) ^1^**	50 ± 3	70 ± 5	X	X	X	X
**·NO (mg/L) ^1^**	1.4 ± 0.3	0.87 ± 0.02	X	X	X	X
**AChE (mg/L) ^2^**	X	X	0.043 ± 0.004	X	X	X
**Tyrosinase (% inh.) ^3^**	X	X	X	X	X	49 ± 6
**XOD (mg/L) ^1^**	X	9 ± 1	X	X	X	X
**Elastase (% inh.) ^4^**	X	X	X	X	53 ± 5	X
**Hyaluronidase (% inh.) ^5^**	<10	<10	X	X	X	X
**Collagenase (% inh.) ^6^**	X	X	X	83 ± 2	X	X

GA: gallic acid; EPI: epicatechin; PHY: physostigmine; PHE: 1, 10-phenanthroline; ELA: elastatinal; KA: Kojic acid; inh.: inhibition. ^1^ Inhibitory Concentration at 50%. ^2^ Inhibitory Concentration at 90%. ^3^ At 21.3 mg/L. ^4^ At 51.26 mg/L. ^5^ From 6 to 220 mg/L. ^6^ At 4500 mg/L.

**Table 5:** Evaluation of platelet-aggregation inhibition of avocado seed and peel against thrombus-formation agonists TRAP-6, ADP and collagen.

Extracts	TRAP-6 (10 μM)		ADP (4 μM)		Collagen (1 μg/mL)	
	PA (%)	Inh. (%)	PA (%)	Inh. (%)	PA (%)	Inh. (%)
**AS**	85 ± 1 ^ns^	0	89 ± 1 ^ns^	3 ± 1	40 ± 6 ***	45 ± 2
**AP**	45 ± 1 ***	42 ± 1	20 ± 2 ***	78 ± 2	32 ± 6 ***	55 ± 2
**Ctrl (−)**	88 ± 1	0	94 ± 1	0	82 ± 3	0
**Ctrl (+)**	22 ± 3	79 ± 2	27 ± 4	69 ± 4	27 ± 1	59 ± 2

Results are expressed as mean ± SEM, *n* = 5. Data were analysed by one-way ANOVA. Post hoc analyses were performed using Dunnet’s test, *** *p* < 0.001 denotes statistically significant differences compared to the negative control (vehicle). ns: denotes nonstatistical differences with respect to the vehicle. ADP: adenosine diphosphate, Inh: inhibition, PA: percentage of platelet aggregation, SEM: standard error, TRAP-6: thrombin-6 receptor-activating peptide.

## Data Availability

All of the data is contained within the article and the Supplementary materials.

## Supplementary Materials

The following supporting information can be downloaded at: https://www.mdpi.com/article/10.3390/antiox11061049/s1, Figure S1: (a) Best peak chromatogram of avocado seed performed by HPLC-ESI-qTOF-MS. The main peaks are numbered as they appear on the table. (b) Best peak chromatogram of avocado peel performed by HPLC-ESI-qTOF-MS. The main peaks are numbered as they appear on the table. (c) Enlarged areas of the avocado-peel chromatogram.

## References

[1] Salazar-López N.J., Domínguez-Avila J.A., Yahia E.M., Belmonte-Herrera B.H., Wall-Medrano A., Montalvo-González E., González-Aguilar G.A. (2020). Avocado fruit and by-products as potential sources of bioactive compounds. Food Res. Int..

[2] Calderón-Oliver M., Escalona-Buendía H.B., Medina-Campos O.N., Pedraza-Chaverri J., Pedroza-Islas R., Ponce-Alquicira E. (2016). Optimization of the antioxidant and antimicrobial response of the combined effect of nisin and avocado byproducts. LWT Food Sci. Technol..

[3] Bhuyan D.J., Alsherbiny M.A., Perera S., Low M., Basu A., Devi O.A., Barooah M.S., Li C.G., Papoutsis K. (2019). The odyssey of bioactive compounds in Avocado (Persea Americana) and their health benefits. Antioxidants.

[4] Velderrain-Rodríguez G.R., Quero J., Osada J., Martín-Belloso O., Rodríguez-Yoldi M.J. (2021). Phenolic-rich extracts from avocado fruit residues as functional food ingredients with antioxidant and antiproliferative properties. Biomolecules.

[5] Rosero J.C., Cruz S., Osorio C., Hurtado N. (2019). Analysis of Phenolic Composition of Byproducts (Seeds and Peels) of Avocado (*Persea americana* Mill.) Cultivated in Colombia. Molecules.

[6] Melgar B., Dias M.I., Ciric A., Sokovic M., Garcia-Castello E.M., Rodriguez-Lopez A.D., Barros L., Ferreira I.C.R.F. (2018). Bioactive characterization of *Persea americana* Mill. by-products: A rich source of inherent antioxidants. Ind. Crops Prod..

[7] Tremocoldi M.A., Rosalen P.L., Franchin M., Massarioli A.P., Denny C., Daiuto É.R., Paschoal J.A.R., Melo P.S., Alencar S.M. (2018). Exploration of avocado by-products as natural sources of bioactive compounds. PLoS ONE.

[8] Ortega-Arellano H.F., Jimenez-Del-Rio M., Velez-Pardo C. (2019). Neuroprotective Effects of Methanolic Extract of Avocado *Persea americana* (var. Colinred) Peel on Paraquat-Induced Locomotor Impairment, Lipid Peroxidation and Shortage of Life Span in Transgenic knockdown Parkin Drosophila melanogaster. Neurochem. Res..

[9] Dabas D., Elias R.J., Ziegler G.R., Lambert J.D. (2019). In Vitro Antioxidant and Cancer Inhibitory Activity of a Colored Avocado Seed Extract. Int. J. Food Sci..

[10] Deuschle V.C.K.N., Brusco I., Piana M., Faccin H., de Carvalho L.M., Oliveira S.M., Viana C. (2018). *Persea americana* Mill. crude extract exhibits antinociceptive effect on UVB radiation-induced skin injury in mice. Inflammopharmacology.

[11] Myung N., Kim S. (2019). The Beneficial Effect of Avocado on Skin Inflammation in a Mouse Model of AD-like Skin Lesions. Korean J. Plant Reources.

[12] 1Laksmiani N.P.L., Sanjaya I.K.N., Leliqia N.P.E. (2020). The activity of avocado (*Persea americana* Mill.) seed extract containing catechin as a skin lightening agent. J. Pharm. Pharmacogn. Res..

[13] Del Castillo-Llamosas A., Rodríguez-Martínez B., del Río P.G., Eibes G., Garrote G., Gullón B. (2021). Hydrothermal treatment of avocado peel waste for the simultaneous recovery of oligosaccharides and antioxidant phenolics. Bioresour. Technol..

[14] Cádiz-Gurrea M.D.L.L., Borrás-Linares I., Lozano-Sánchez J., Joven J., Fernández-Arroyo S., Segura-Carretero A. (2017). Cocoa and grape seed byproducts as a source of antioxidant and anti-inflammatory proanthocyanidins. Int. J. Mol. Sci..

[15] De La Luz Cádiz-Gurrea M., Fernández-Ochoa Á., Leyva-Jiménez F.J., Guerrero-Muñoz N., Del Carmen Villegas-Aguilar M., Pimentel-Moral S., Ramos-Escudero F., Segura-Carretero A. (2020). LC-MS and spectrophotometric approaches for evaluation of bioactive compounds from Peru cocoa by-products for commercial applications. Molecules.

[16] Re R., Pellegrini N., Proteggente A., Pannala A., Yang M., Rice-Evans C. (1999). Antioxidant activity applying an improved ABTS radical cation decolorization assay. Free Radic. Biol. Med..

[17] Huang D., Ou B., Hampsch-Woodill M., Flanagan J.A., Prior R.L. (2002). High-throughput assay of oxygen radical absorbance capacity (ORAC) using a multichannel liquid handling system coupled with a microplate fluorescence reader in 96-well format. J. Agric. Food Chem..

[18] Gomes A., Fernandes E., Silva A.M.S., Santos C.M.M., Pinto D.C.G.A., Cavaleiro J.A.S., Lima J.L.F.C. (2007). 2-Styrylchromones: Novel strong scavengers of reactive oxygen and nitrogen species. Bioorganic Med. Chem..

[19] Pinto D., De La Luz Cádiz-Gurrea M., Sut S., Ferreira A.S., Leyva-Jimenez F.J., Dall’acqua S., Segura-Carretero A., Delerue-Matos C., Rodrigues F. (2020). Valorisation of underexploited Castanea sativa shells bioactive compounds recovered by supercritical fluid extraction with CO_2_: A response surface methodology approach. J. CO2 Util..

[20] Ellman G.L., Courtney K.D., Andres V., Featherstone R.M. (1961). A new and rapid colorimetric determination of acetylcholinesterase activity. Biochem. Pharmacol..

[21] Pinto D., Cádiz-Gurrea M.L., Garcia J., Saavedra M.J., Freitas V., Costa P., Sarmento B., Delerue-Matos C., Rodrigues F. (2021). From soil to cosmetic industry: Validation of a new cosmetic ingredient extracted from chestnut shells. Sustain. Mater. Technol..

[22] Nema N.K., Maity N., Sarkar B., Mukherjee P.K. (2010). Cucumis sativus fruit-potential antioxidant, anti-hyaluronidase, and anti-elastase agent. Arch. Dermatol. Res..

[23] Nema N.K., Maity N., Sarkar B.K., Mukherjee P.K. (2013). Matrix metalloproteinase, hyaluronidase and elastase inhibitory potential of standardized extract of *Centella asiatica*. Pharm. Biol..

[24] Rickham P.P. (1964). Human Experimentation: Code of Ethics of the World Medical Association. Br. Med. J..

[25] Rodríguez L. (2021). Antiplatelet Effect of Aristotelia Chilensis (Maqui) Extracts Through In Vitro Studies.

[26] Rodríguez L., Badimon L., Méndez D., Padró T., Vilahur G., Peña E., Carrasco B., Vogel H., Palomo I., Fuentes E. (2021). Antiplatelet Activity of Isorhamnetin via Mitochondrial Regulation. Antioxidants.

[27] Rojas-Garbanzo C., Rodríguez L., Pérez A.M., Mayorga-Gross A.L., Vásquez-Chaves V., Fuentes E., Palomo I. (2021). Anti-platelet activity and chemical characterization by UPLC-DAD-ESI-QTOF-MS of the main polyphenols in extracts from Psidium leaves and fruits. Food Res. Int..

[28] Fuentes E., Badimon L., Caballero J., Padró T., Vilahur G., Alarcón M., Pérez P., Palomo I. (2014). Protective mechanisms of adenosine 5′-monophosphate in platelet activation and thrombus formation. Thromb. Haemost..

[29] Alarcón M., Bustos M., Mendez D., Fuentes E., Palomo I., Lutz M. (2020). In Vitro Assay of Quinoa (*Chenopodium quinoa* Willd.) and Lupin (*Lupinus* spp.) Extracts on Human Platelet Aggregation. Plant Foods Hum. Nutr..

[30] Figueroa J.G., Borrás-Linares I., Lozano-Sánchez J., Segura-Carretero A. (2018). Comprehensive characterization of phenolic and other polar compounds in the seed and seed coat of avocado by HPLC-DAD-ESI-QTOF-MS. Food Res. Int..

[31] Figueroa J.G., Borrás-Linares I., Lozano-Sánchez J., Segura-Carretero A. (2018). Comprehensive identification of bioactive compounds of avocado peel by liquid chromatography coupled to ultra-high-definition accurate-mass Q-TOF. Food Chem..

[32] Trujillo-Mayol I., Casas-Forero N., Pastene-Navarrete E., Silva F.L., Alarcón-Enos J. (2021). Fractionation and hydrolyzation of avocado peel extract: Improvement of antibacterial activity. Antibiotics.

[33] López-Cobo A., Gómez-Caravaca A.M., Pasini F., Caboni M.F., Segura-Carretero A., Fernández-Gutiérrez A. (2016). HPLC-DAD-ESI-QTOF-MS and HPLC-FLD-MS as valuable tools for the determination of phenolic and other polar compounds in the edible part and by-products of avocado. LWT Food Sci. Technol..

[34] Del Refugio Ramos M., Jerz G., Villanueva S., López-Dellamary F., Waibel R., Winterhalter P. (2004). Two glucosylated abscisic acid derivates from avocado seeds (*Persea americana* Mill. Lauraceae cv. Hass). Phytochemistry.

[35] Figueroa J.G., Borrás-Linares I., Del Pino-García R., Curiel J.A., Lozano-Sánchez J., Segura-Carretero A. (2021). Functional ingredient from avocado peel: Microwave-assisted extraction, characterization and potential applications for the food industry. Food Chem..

[36] Pei H., Su W., Gui M., Dou M., Zhang Y., Wang C., Lu D. (2021). Comparative analysis of chemical constituents in different parts of lotus by UPLC and QToF-MS. Molecules.

[37] Ouyang M.-A., Wein Y., Kuo Y. (2007). Four New Lariciresinol-Based Lignan Glycosides from the Roots of Rhus javanica var.roxburghiana. Helv. Chim. Acta.

[38] Yu Y.T., Wei X., Liu Y., Dong G., Hao C.Y., Zhang J., Jiang J.Z., Cheng J.T., Liu A., Chen S. (2022). Identification and quantification of oligomeric proanthocyanidins, alkaloids, and flavonoids in lotus seeds: A potentially rich source of bioactive compounds. Food Chem..

[39] Golukcu M., Ozdemir F. (2010). Changes in phenolic composition of avocado cultivars during harvesting time. Chem. Nat. Compd..

[40] Wang W., Bostic T.R., Gu L. (2010). Antioxidant capacities, procyanidins and pigments in avocados of different strains and cultivars. Food Chem..

[41] Dabas D., Shegog R.M., Ziegler G.R., Lambert J.D. (2013). Avocado (*Persea americana*) Seed as a Source of Bioactive Phytochemicals. Curr. Pharm. Des..

[42] Sánchez-Quezada V., Campos-Vega R., Loarca-Piña G. (2021). Prediction of the Physicochemical and Nutraceutical Characteristics of ‘Hass’ Avocado Seeds by Correlating the Physicochemical Avocado Fruit Properties According to Their Ripening State. Plant Foods Hum. Nutr..

[43] Akan S. (2021). Phytochemicals in avocado peel and their potential uses. Food Health.

[44] Cádiz-Gurrea M.L., Fernández-Arroyo S., Segura-Carretero A. (2014). Pine bark and green tea concentrated extracts: Antioxidant activity and comprehensive characterization of bioactive compounds by HPLC-ESI-QTOF-MS. Int. J. Mol. Sci..

[45] Munteanu I.G., Apetrei C. (2021). Analytical methods used in determining antioxidant activity: A review. Int. J. Mol. Sci..

[46] Kosińska A., Karamać M., Estrella I., Hernández T., Bartolomé B., Dykes G.A. (2012). Phenolic compound profiles and antioxidant capacity of *Persea americana* mill. peels and seeds of two varieties. J. Agric. Food Chem..

[47] Ben-Othman S., Jõudu I., Bhat R. (2020). Bioactives from Agri-Food Wastes: Present Insights and Future Challenges. Molecules.

[48] Oboh G., Odubanjo V.O., Bello F., Ademosun A.O., Oyeleye S.I., Nwanna E.E., Ademiluyi A.O. (2016). Aqueous extracts of avocado pear (*Persea americana* Mill.) leaves and seeds exhibit anti-cholinesterases and antioxidant activities in vitro. J. Basic Clin. Physiol. Pharmacol..

[49] Trujillo-Mayol I., Badillo-Muñoz G., Céspedes-Acuña C., Alarcón-Enos J. (2020). The relationship between fruit size and phenolic and enzymatic composition of avocado byproducts (*Persea americana* mill.): The importance for biorefinery applications. Horticulturae.

[50] Rodríguez-Carpena J.G., Morcuende D., Andrade M.J., Kylli P., Estevez M. (2011). Avocado (*Persea americana* Mill.) phenolics, In Vitro antioxidant and antimicrobial activities, and inhibition of lipid and protein oxidation in porcine patties. J. Agric. Food Chem..

[51] Dibacto R.E.K., Tchuente B.R.T., Nguedjo M.W., Tientcheu Y.M.T., Nyobe E.C., Edoun F.L.E., Kamini M.F.G., Dibanda R.F., Medoua G.N. (2021). Total Polyphenol and Flavonoid Content and Antioxidant Capacity of Some Varieties of *Persea americana* Peels Consumed in Cameroon. Sci. World J..

[52] Alañón M.E., Pimentel-Moral S., Arráez-Román D., Segura-Carretero A. (2021). HPLC-DAD-Q-ToF-MS profiling of phenolic compounds from mango (*Mangifera indica* L.) seed kernel of different cultivars and maturation stages as a preliminary approach to determine functional and nutraceutical value. Food Chem..

[53] Santos W.N.L., da Silva Sauthier M.C., dos Santos A.M.P., de Andrade Santana D., Almeida Azevedo R.S., da Cruz Caldas J. (2017). Simultaneous determination of 13 phenolic bioactive compounds in guava (*Psidium guajava* L.) by HPLC-PAD with evaluation using PCA and Neural Network Analysis (NNA). Microchem. J..

[54] Rodrigues F., Palmeira-de-Oliveira A., das Neves J., Sarmento B., Amaral M.H., Oliveira M.B. (2013). Medicago spp. extracts as promising ingredients for skin care products. Ind. Crops Prod..

[55] Huang D., Boxin O.U., Prior R.L. (2005). The chemistry behind antioxidant capacity assays. J. Agric. Food Chem..

[56] Zeng Y., Song J., Zhang M., Wang H., Zhang Y., Suo H. (2020). Comparison of in vitro and in vivo antioxidant activities of six flavonoids with similar structures. Antioxidants.

[57] Rice-Evans C.A., Miller N.J., Paganga G. (1996). Structure-antioxidant activity relationships of flavonoids and phenolic acids. Free Radic. Biol. Med..

[58] Veiko A.G., Lapshina E.A., Zavodnik I.B. (2021). Comparative analysis of molecular properties and reactions with oxidants for quercetin, catechin, and naringenin. Mol. Cell. Biochem..

[59] Boots A.W., Haenen G.R.M.M., Bast A. (2008). Health effects of quercetin: From antioxidant to nutraceutical. Eur. J. Pharmacol..

[60] Song X., Wang Y., Gao L. (2020). Mechanism of antioxidant properties of quercetin and quercetin-DNA complex. J. Mol. Model..

[61] Amado D.A.V., Helmann G.A.B., Detoni A.M., de Carvalho S.L.C., de Aguiar C.M., Martin C.A., Tiuman T.S., Cottica S.M. (2019). Antioxidant and antibacterial activity and preliminary toxicity analysis of four varieties of avocado (*Persea americana* Mill.). Brazilian J. Food Technol..

[62] Segovia F.J., Hidalgo G.I., Villasante J., Ramis X., Almajano M.P. (2018). Avocado seed: A comparative study of antioxidant content and capacity in protecting oil models from oxidation. Molecules.

[63] Castañeda-Valbuena D., Ayora-Talavera T., Luján-Hidalgo C., Álvarez-Gutiérrez P., Martínez-Galero N., Meza-Gordillo R. (2021). Ultrasound extraction conditions effect on antioxidant capacity of mango by-product extracts. Food Bioprod. Process..

[64] Morais D.R., Rotta E.M., Sargi S.C., Schmidt E.M., Bonafe E.G., Eberlin M.N., Sawaya A.C.H.F., Visentainer J.V. (2015). Antioxidant activity, phenolics and UPLC-ESI(-)-MS of extracts from different tropical fruits parts and processed peels. Food Res. Int..

[65] Flaczyk E., Kobus-Cisowska J., Przeor M., Korczak J., Remiszewski M., Korbas E., Buchowski M. (2013). Chemical characterization and antioxidative properties of Polish variety of Morus alba L. leaf aqueous extracts from the laboratory and pilot-scale processes. Agric. Sci..

[66] Alagbaoso C.A., Tokunbo I.I., Osakwe O.S. (2015). Comparative Study of Antioxidant Activity and Mineral Composition of Methanol Extract of Seeds of Ripe and Unripe Avocado Pear (*Persea americana*, Mill.). NISEB J..

[67] Rao S.B., Jayanthi M., Yogeetha R., Ramakrishnaiah H., Nataraj J. (2013). Free radical scavenging activity and reducing power of gnidia glauca(fresen.) gilg. J. Appl. Pharm. Sci..

[68] Pietta P.G. (2000). Flavonoids as antioxidants. J. Nat. Prod..

[69] Kamaraj M., Dhana Rangesh Kumar V., Nithya T.G., Danya U. (2019). Assessment of Antioxidant, Antibacterial Activity and Phytoactive Compounds of Aqueous Extracts of Avocado Fruit Peel from Ethiopia. Int. J. Pept. Res. Ther..

[70] Laver J.R., Stevanin T.M., Read R.C. (2008). Chemiluminescence Quantification of NO and Its Derivatives in Liquid Samples. Methods Enzymol..

[71] Nabavi S.F., Nabavi S.M., Setzer W., Nabavi S.A., Nabavi S.A., Ebrahimzadeh M.A. (2013). Antioxidant and antihemolytic activity of lipid-soluble bioactive substances in avocado fruits. Fruits.

[72] Melo P.S., Massarioli A.P., Denny C., Dos Santos L.F., Franchin M., Pereira G.E., Vieira T.M.F.D.S., Rosalen P.L., De Alencar S.M. (2015). Winery by-products: Extraction optimization, phenolic composition and cytotoxic evaluation to act as a new source of scavenging of reactive oxygen species. Food Chem..

[73] Andjelković M., Van Camp J., De Meulenaer B., Depaemelaere G., Socaciu C., Verloo M., Verhe R. (2006). Iron-chelation properties of phenolic acids bearing catechol and galloyl groups. Food Chem..

[74] Hapner C.D., Deuster P., Chen Y. (2010). Inhibition of oxidative hemolysis by quercetin, but not other antioxidants. Chem. Biol. Interact..

[75] López-López G., Moreno L., Cogolludo A., Galisteo M., Ibarra M., Duarte J., Lodi F., Tamargo J., Perez-Vizcaino F. (2004). Nitric Oxide (NO) Scavenging and NO Protecting Effects of Quercetin and Their Biological Significance in Vascular Smooth Muscle. Mol. Pharmacol..

[76] Priya Darsini D.T., Maheshu V., Vishnupriya M., Nishaa S., Sasikumar J.M. (2013). Antioxidant potential and amino acid analysis of underutilized tropical fruit *Limonia acidissima* L.. Free Radicals Antioxid..

[77] Rymbai H., Srivastav M., Sharma R.R., Patel C.R., Singh A.K. (2013). Bio-active compounds in mango (*Mangifera indica* L.) and their roles in human health and plant defence-A review. J. Hortic. Sci. Biotechnol..

[78] Aazza S., Lyoussi B., Miguel M.G. (2011). Antioxidant and antiacetylcholinesterase activities of some commercial essential oils and their major compounds. Molecules.

[79] Boutoub O., El-Guendouz S., Manhita A., Dias C.B., Estevinho L.M., Paula V.B., Carlier J., Costa M.C., Rodrigues B., Raposo S. (2021). Comparative Study of the Antioxidant and Enzyme Inhibitory Activities of Two Types of Moroccan Euphorbia Entire Honey and Their Phenolic Extracts. Foods.

[80] Di Petrillo A., Santos-Buelga C., Era B., González-Paramás A.M., Tuberoso C.I.G., Medda R., Pintus F., Fais A. (2017). Sardinian honeys as sources of xanthine oxidase and tyrosinase inhibitors. Food Sci. Biotechnol..

[81] Cádiz-Gurrea M.L., Pinto D., Delerue-Matos C., Rodrigues F. (2021). Olive fruit and leaf wastes as bioactive ingredients for cosmetics—A preliminary study. Antioxidants.

[82] Pientaweeratch S., Panapisal V., Tansirikongkol A. (2016). Antioxidant, anti-collagenase and anti-elastase activities of Phyllanthus emblica, Manilkara zapota and silymarin: An In Vitro comparative study for anti-aging applications. Pharm. Biol..

[83] Wittenauer J., MäcKle S., Sußmann D., Schweiggert-Weisz U., Carle R. (2015). Inhibitory effects of polyphenols from grape pomace extract on collagenase and elastase activity. Fitoterapia.

[84] Kanashiro A., Souza J.G., Kabeya L.M., Azzolini A.E.C.S., Lucisano-Valim Y.M. (2007). Elastase release by stimulated neutrophils inhibited by flavonoids: Importance of the catechol group. Z. Fur Naturforsch. Sect. C J. Biosci..

[85] Sin B.Y., Kim H.P. (2005). Inhibition of collagenase by naturally-occurring flavonoids. Arch. Pharm. Res..

[86] Fan M., Zhang G., Hu X., Xu X., Gong D. (2017). Quercetin as a tyrosinase inhibitor: Inhibitory activity, conformational change and mechanism. Food Res. Int..

[87] Schmitt M., Alabdul Magid A., Hubert J., Etique N., Duca L., Voutquenne-Nazabadioko L. (2020). Bio-guided isolation of new phenolic compounds from Hippocrepis emerus flowers and investigation of their antioxidant, tyrosinase and elastase inhibitory activities. Phytochem. Lett..

[88] Polat Kose L., Bingol Z., Kaya R., Goren A.C., Akincioglu H., Durmaz L., Koksal E., Alwasel S.H., Gülçin İ. (2020). Anticholinergic and antioxidant activities of avocado (*Folium perseae*) leaves–phytochemical content by LC-MS/MS analysis. Int. J. Food Prop..

[89] Fawole O.A., Makunga N.P., Opara U.L. (2012). Antibacterial, antioxidant and tyrosinase-inhibition activities of pomegranate fruit peel methanolic extract. BMC Complement. Altern. Med..

[90] Amis R.T., Novalinda Ginting C., Ferdinand S., Ikhtiari R. Anti-hyperuricemia of Avocado Leaves Ethanol Extract in Potassium Oxonate Induced-Rats. Proceedings of the 2021 IEEE International Conference on Health, Instrumentation & Measurement, and Natural Sciences.

[91] Samejima H., Park B. (2019). Inhibition Activity of Guava (*Psidium guajava* L.) Leaf Extract against Collagenase, Elastase, Hyaluronidase, and Carbohydrate Digestion Enzymes. Trop. Agric. Dev..

[92] Ochocka R., Hering A., Stefanowicz-Hajduk J., Cal K., Barańska H. (2017). The effect of mangiferin on skin: Penetration, permeation and inhibition of ECM enzymes. PLoS ONE.

[93] Pineda-Lozano J.E., Martínez-Moreno A.G., Virgen-Carrillo C.A. (2021). The Effects of Avocado Waste and Its Functional Compounds in Animal Models on Dyslipidemia Parameters. Front. Nutr..

[94] Sharifi-Rad J., Quispe C., Shaheen S., El Haouari M., Azzini E., Butnariu M., Sarac I., Pentea M., Ramírez-Alarcón K., Martorell M. (2021). Flavonoids as potential anti-platelet aggregation agents: From biochemistry to health promoting abilities. Crit. Rev. Food Sci. Nutr..

[95] Fuentes E., Gibbins J.M., Holbrook L.M., Palomo I. (2018). NADPH oxidase 2 (NOX2): A key target of oxidative stress-mediated platelet activation and thrombosis. Trends Cardiovasc. Med..

[96] Schick B.P. (2010). Serglycin Proteoglycan Deletion in Mouse Platelets: Physiological Effects and their Implications for Platelet Contributions to Thrombosis, Inflammation, Atherosclerosis, and Metastasis.

[97] Kobarfard F., Ayatollahi S.A., Khosravi-Dehaghi N., Faizi M., Amidi S., Martorell M., Choudhary M.I., Suleria H.A.R., Sharifi-Rad J. (2020). High-performance thin-layer chromatography fingerprinting, total phenolic and total flavonoid contents and anti-platelet-aggregation activities of Prosopis farcta extracts. Cell. Mol. Biol..

[98] Méndez D., Urra F.A., Millas-Vargas J.P., Alarcón M., Rodríguez-Lavado J., Palomo I., Trostchansky A., Araya-Maturana R., Fuentes E. (2020). Synthesis of antiplatelet ortho-carbonyl hydroquinones with differential action on platelet aggregation stimulated by collagen or TRAP-6. Eur. J. Med. Chem..

[99] Olas B. (2021). A review of in vitro studies of the anti-platelet potential of citrus fruit flavonoids. Food Chem. Toxicol..

