# Supplementary material for: Biological Evaluation of Avocado Residues as a Potential Source of Bioactive Compounds
Source: Antioxidants (Basel). 2022 May 25;11(6):1049. doi: 10.3390/antiox11061049 (PMC9220077; doi:10.3390/antiox11061049)
Supplement: Supplementary file 1 [file antioxidants-11-01049-s001.zip › antioxidants-1725313-supplementary.pdf]

SUPPLEMENTARY MATERIALS

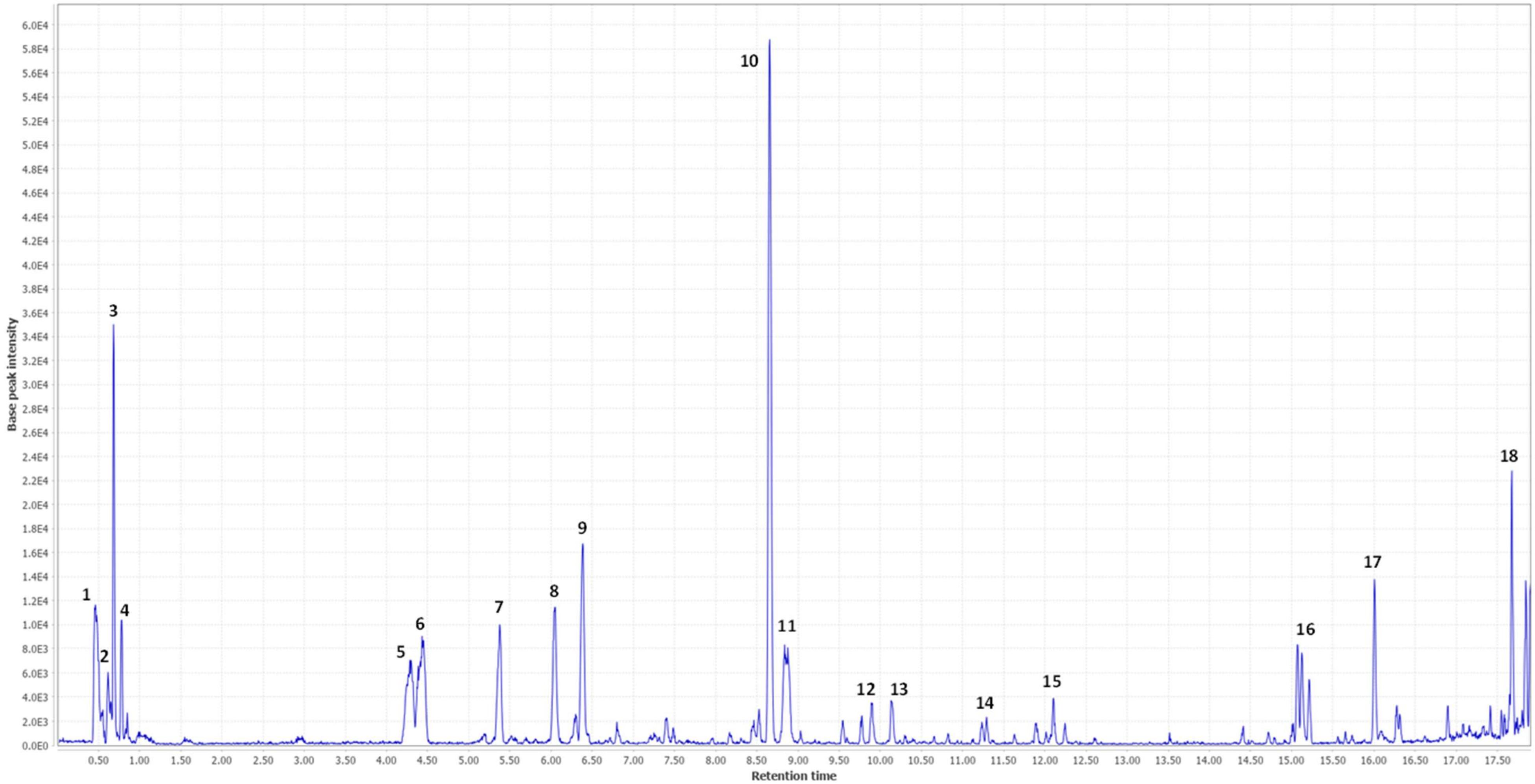

Figure S1. (a) Best peak chromatogram of avocado seed performed by HPLC-ESI-qTOF-MS. The main peaks are numbered as they appear on the table.

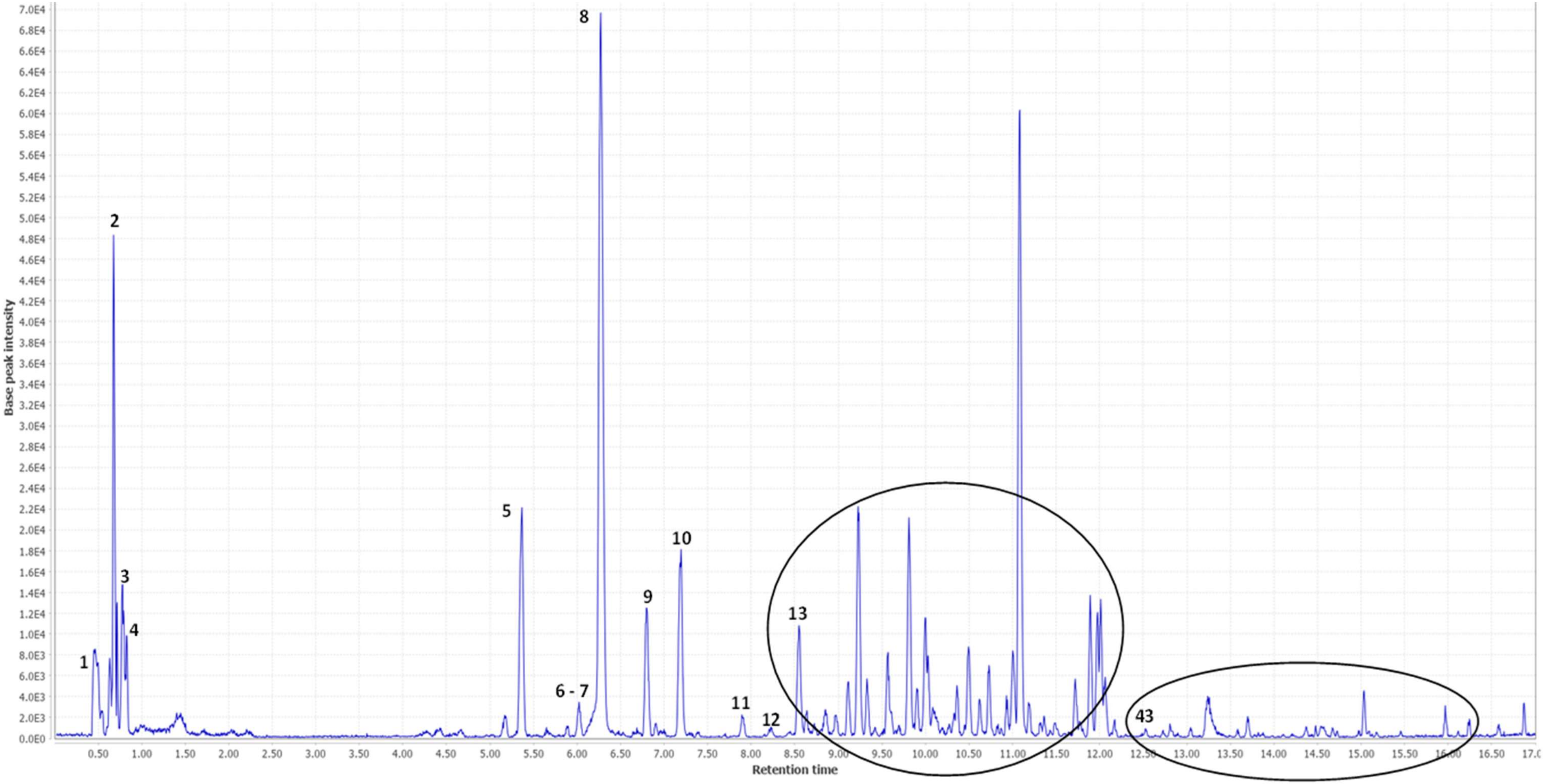

Figure S1. (b) Best peak chromatogram of avocado peel performed by HPLC-ESI-qTOF-MS. The main peaks are numbered as they appear on the table.

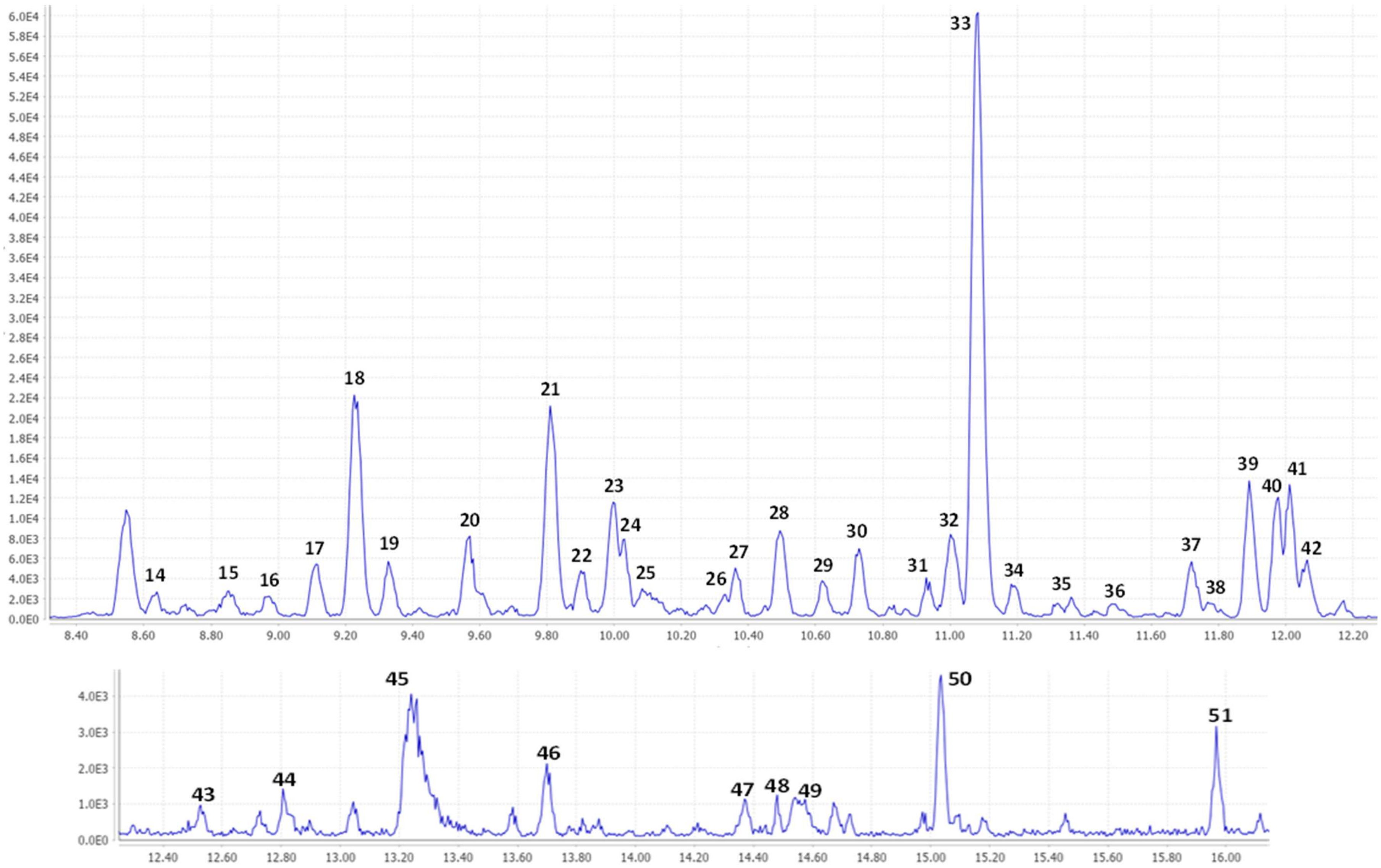

Figure S1. (c) Enlarged areas of the avocado peel chromatogram.
